# Anchored Phylogenomics, Evolution and Systematics of Elateridae: Are All Bioluminescent Elateroidea Derived Click Beetles?

**DOI:** 10.3390/biology10060451

**Published:** 2021-05-21

**Authors:** Hume B. Douglas, Robin Kundrata, Adam J. Brunke, Hermes E. Escalona, Julie T. Chapados, Jackson Eyres, Robin Richter, Karine Savard, Adam Ślipiński, Duane McKenna, Jeremy R. Dettman

**Affiliations:** 1 Agriculture and Agri-Food Canada, 960 Carling Avenue, Ottawa, ON K1A 0C6, Canada; adam.brunke@canada.ca (A.J.B.); julie.chapados@canada.ca (J.T.C.); jackson.eyres@canada.ca (J.E.); robin.richter@canada.ca (R.R.); karine.savard@canada.ca (K.S.); jeremy.dettman@canada.ca (J.R.D.); 2 Department of Zoology, Faculty of Science, Palacky University, 17. listopadu 50, 771 46 Olomouc, Czech Republic; robin.kundrata@upol.cz; 3 Australian National Insect Collection, National Collections Australia, CSIRO, Canberra, ACT 2601, Australia; hermes.escalona@csiro.au (H.E.E.); Adam.Slipinski@csiro.au (A.Ś.); 4 Center for Biodiversity Research, Department of Biological Sciences, University of Memphis, Memphis, TN 38152, USA; dmckenna@memphis.edu

**Keywords:** anchored hybrid enrichment, baitset, classification, Elateridae, four-cluster likelihood mapping, Lampyridae, Phengodidae, phylogenomics, Rhagophthalmidae, Sinopyrophoridae

## Abstract

**Simple Summary:**

In the era of phylogenomics, new molecular sequencing and computational techniques can aid in resolving phylogenetic relationships that were previously intractable by morphological or limited molecular data. In this study, we used anchored hybrid enrichment—designed to recover DNA sequences from hundreds of single-copy orthologous genes—to resolve the phylogeny of the Elateridae (click-beetles) and establish their placement within superfamily Elateroidea. The resulting data were compatible with published transcriptomes, allowing for integrating our dataset with previously published data. Using a wide range of analyses on these molecular data, we tested hypotheses long-debated in the morphological literature and also the robustness of our phylogenetic inferences. Our results placed the bioluminescent lampyroids (fireflies and relatives) within the click-beetles, challenging the current classification of Elateridae, Lampyridae, Phengodidae, and Rhagophthalmidae. However, despite the large amount of molecular data analyzed, a few nodes with conflicting phylogenetic signals could not be unambiguously resolved. Overall, we recovered well-resolved tree topologies that will serve as a framework for further systematic and evolutionary studies of click-beetles. This work further demonstrates that the click-beetle lineage contains not only pest wireworms, but also many species that benefit agriculture.

**Abstract:**

Click-beetles (Coleoptera: Elateridae) are an abundant, diverse, and economically important beetle family that includes bioluminescent species. To date, molecular phylogenies have sampled relatively few taxa and genes, incompletely resolving subfamily level relationships. We present a novel probe set for anchored hybrid enrichment of 2260 single-copy orthologous genes in Elateroidea. Using these probes, we undertook the largest phylogenomic study of Elateroidea to date (99 Elateroidea, including 86 Elateridae, plus 5 non-elateroid outgroups). We sequenced specimens from 88 taxa to test the monophyly of families, subfamilies and tribes. Maximum likelihood and coalescent phylogenetic analyses produced well-resolved topologies. Notably, the included non-elaterid bioluminescent families (Lampyridae + Phengodidae + Rhagophthalmidae) form a clade within the otherwise monophyletic Elateridae, and Sinopyrophoridae may not warrant recognition as a family. All analyses recovered the elaterid subfamilies Elaterinae, Agrypninae, Cardiophorinae, Negastriinae, Pityobiinae, and Tetralobinae as monophyletic. Our results were conflicting on whether the hypnoidines are sister to Dendrometrinae or Cardiophorinae + Negastriinae. Moreover, we show that fossils with the eucnemid-type frons and elongate cylindrical shape may belong to Eucnemidae, Elateridae: Thylacosterninae, ancestral hard-bodied cantharoids or related extinct groups. Proposed taxonomic changes include recognition of Plastocerini as a tribe in Dendrometrinae and Hypnoidinae stat. nov. as a subfamily within Elateridae.

## 1. Introduction

**Figure 1:**
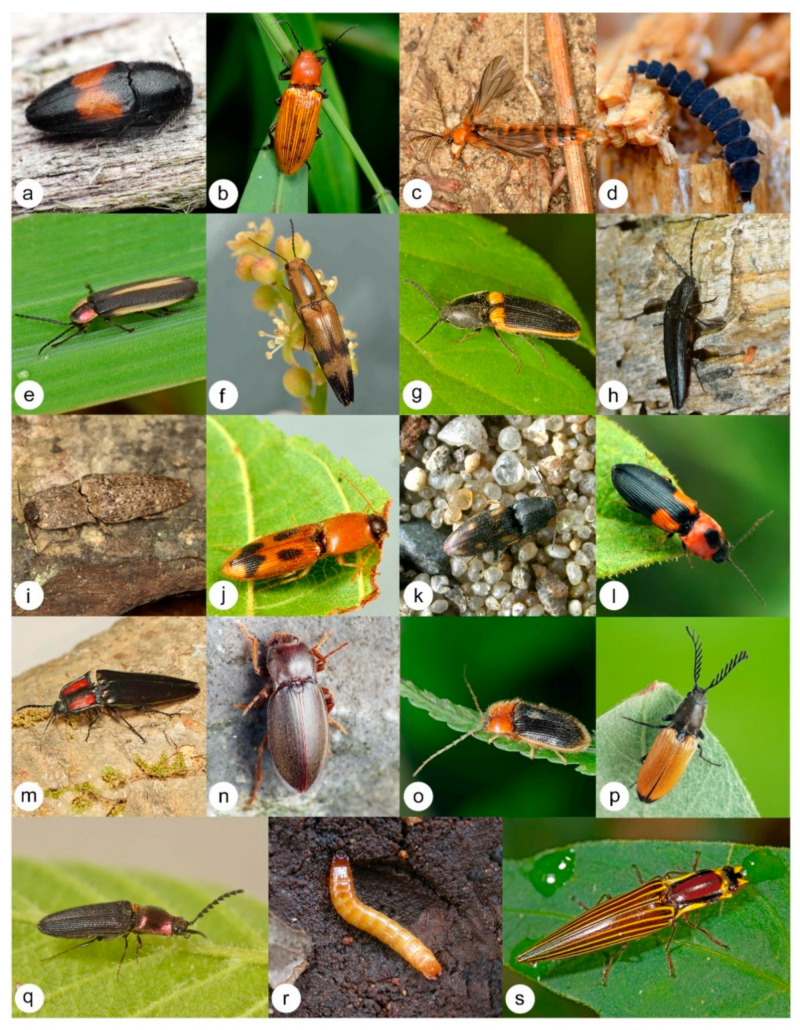
Representatives of the subfamilies of Elateridae and the lampyroid clade (**a**) *Drapetes mordelloides* (Host, 1789) (Lissominae; image: T. Németh), (**b**) *Hemiops* sp. (Hemiopinae; image: S. A. Marshall), (**c**) *Phengodes* sp. (Phengodidae; image: S. A. Marshall), (**d**) *Lampyris* sp. (Lampyridae; image: A. Prosvirov), (**e**) Lampyridae (image: S. A. Marshall), (**f**) *Aphanopenthes vanus* (Germar, 1844) (Elaterinae; image: S. A. Marshall), (**g**) *Ampedus oblessus* (Say, 1833) (Elaterinae; image: S. A. Marshall), (**h**) *Tibionema* sp. (Pityobiinae; image: S. A. Marshall), (**i**) *Cryptalaus* sp. (Agrypninae; image: F. Trnka), (**j**) *Aeolus* sp. (Agrypninae; image: S. A. Marshall), (**k**) *Negastrius pulchellus* (Linnaeus, 1761) (Negastriinae; image: www.elateridae.com accessed on 8 January 2021), (**l**) *Cardiophorus kindermanni* Candèze, 1860 (Cardiophorinae; image: T. Németh), (**m**) *Campsosternus* sp. (Dendrometrinae; image: S. A. Marshall), (**n**) *Dima pelikani* Mertlik, Németh and Kundrata, 2017 (Dendrometrinae; image: T. Németh), (**o**) *Penia cinctipennis* Fleutiaux, 1936 (Dendrometrinae; image: S. A. Marshall), (**p**) *Anostirus castaneus* (Linnaeus, 1758) (Dendrometrinae; image: T. Németh), (**q**) *Limonius aurifer* (LeConte, 1853) (Dendrometrinae; image: S. A. Marshall), (**r**) *Gambrinus violaceus* (Müller, 1821) (Dendrometrinae; image: www.elateridae.com accessed on 8 January 2021), (**s**) *Semiotus bispinus* Candèze, 1874 (Dendrometrinae; image: S. A. Marshall).

Elateridae, with over 11,000 species distributed in all zoogeographical regions, are among the largest and most diverse beetle families (Figure 1), and elaterid species collectively exhibit a diversity of trophic habits—perhaps more than any other beetle family [1]. Their crown group and taxonomic diversification can be traced back to the Mesozoic, although their precise age remains uncertain [2,3,4,5,6]. Members include economically significant agricultural pests, fungivores, beneficial snail predators, sub-cortical predators, predators of vertebrate eggs, termitophiles, myrmecophiles, and forest, desert and semi-aquatic species [7]. The classification of Elateridae is in disarray with several conflicting systems, which contributes to the challenge of taxonomic and applied research on its many poorly known species. Classification and biological understanding of Elateridae is limited by a lack of phylogenetic resolution and the dozens of subfamily names used in recent classifications.

Efforts to resolve the subfamily level phylogeny of click-beetles using morphology [8,9] have had little success. Phylogenies using few genes have recovered some subfamilies as classically defined and have provided new insights [10,11,12,13,14,15]. However, these studies often lacked resolution or statistical support for subfamily level relationships, and morphological studies omitted exemplars of elateroid families now known to belong within Elateridae. These include Drilidae [10], Omalisidae [16], and Plastoceridae [14]. Newer phylogenomic studies of Elateroidea using data from next-generation sequencing technologies have yielded higher branch support values at basal nodes but have included relatively few Elateridae [2,3,6,16,17]. Moreover, they also raise further questions about the monophyly of Elateridae.

Kusy et al. [6] applied data-rich phylogenomic evidence to resolve the phylogeny of Elateroidea. They recovered the recently discovered bioluminescent elaterid *Sinopyrophorus* Bi and Li, 2019 as a close relative of their “lampyroid clade” (Phengodidae, Rhagophthalmidae, and Lampyridae, together with over 2500 species). Interpreting their results to mean that *Sinopyrophorus* was not part of Elateridae, they erected the new family Sinopyrophoridae. However, their study recovered conflicting tree topologies according to different analytical approaches and sampled only 22 species of Elateridae and 24 lampyroids. These shortcomings limited their study’s ability to test the monophyly of Elateridae and whether *Sinopyrophorus* is separate from Elateridae or derived from within it.

Despite these limitations, analyses of existing datasets have recovered several consistent relationships. Most DNA studies found some or all lampyroids as sister to Elateridae ([6] in part, [12,14] in part, [16]), or part of Elateridae ([2,3,6] in part). Both morphological and molecular studies agree on the monophyly of Cardiophorinae and Cardiophorinae + Negastriinae [9,12,15,18] and on the monophyly of Agrypninae [9,10,11,12,13,15,19], including former Drilidae when studied. The largest subfamily, Elaterinae, are monophyletic in all DNA-based studies [6,10,13,14,20] with incorporation of Eudicronychinae, where tested. The monophyly of subfamily Dendrometrinae has been challenged by recent authors, e.g., [12,13], even after incorporation of subfamilies Oxynopterinae and Semiotinae [10] and demonstration that family Plastoceridae should also be included [14].

Morphology and DNA-based phylogenies also find conflict between some well-supported clades. Douglas’ [9] morphological phylogeny recovered Hypnoidini as sister to Negastriinae + Cardiophorinae, whereas 4-gene DNA nucleotide-based trees placed them in Dendrometrinae [12], with Negastriinae + Cardiophorinae possibly also a part of a widely defined Dendrometrinae clade. Douglas [9] also found Tetralobinae as sister to Agrypninae, agreeing with classical taxonomies but disagreeing with a phylogeny based on nucleotide sequence data from four genes by Kundrata et al. [13].

Resolution of these problematic deeper nodes may be possible using anchored hybrid enrichment (AHE), which uses short DNA sequences as baits to target hundreds to thousands of single-copy, orthologous nuclear loci across a wide range of insects [21,22,23,24], including Elateroidea [17]. Here we have developed a novel AHE probe set to produce molecular phylogenetic datasets for the Elateroidea. We demonstrate that this probe set captures phylogenetic signal within the Elateroidea, focusing particularly on Elateridae and their close lampyroid relatives. The resulting phylogenomic dataset for Elateroidea and Elateridae is the largest produced. We used it to test the monophyly of family Elateridae, its major subfamilies, and the phylogenetic relationships of the elaterid subfamilies and tribes.

## 2. Materials and Methods

### 2.1. Taxon Sampling

Since the Elateridae have consistently been recovered within a well-supported Elateroidea [2,3,6,11,14], all taxa were sampled from Elateroidea and Elateridae. Additional Elateriformia, i.e., Buprestidae: *Ptosima undecimmaculata* (Herbst, 1784), Byrrhidae: *Byrrhus* sp. and *Notolioon* sp., Dryopidae: *Dryops* sp., and Heteroceridae: *Heterocerus fenestratus* (Thunberg, 1784)), were used as outgroups (*Ptosima* used for tree rooting). Dried museum specimens were used for *Tibionema abdominalis* Guérin-Méneville, 1838 and Lycidae sp. (Supplementary Table S1). The classification of Elateridae follows Kundrata et al. [13] and Kusy et al. [16,19]. Classifications of other families follow Bouchard et al. [25], except for the incorporation of Omalisidae [16], Drilidae [10], and Plastoceridae into Elateridae [14]. The final 104-taxon dataset included 93 newly sequenced taxa and 11 published transcriptomes [2], from which target loci were extracted. We sampled Elateridae most densely (Supplementary Table S1), including representatives from 13 of 19 described extant subfamilies. Elaterid subfamilies not represented include Campyloxeninae, Morostominae, Parablacinae, Physodactylinae, Subprotelaterinae, and Thylacosterninae, which together represent less than one percent of described elaterid species diversity. Of these, Morostominae may belong in Dendrometrinae [15], species of *Physodactylus* Fischer von Waldheim, 1823 are most likely fossorial Elaterinae [26], and Thylacosterninae cluster consistently with Lissominae [11,12,15]. At the tribal level, we sampled 27 of 42 elaterid tribes (Supplementary Table S1).

### 2.2. ElaterBaits Probe Design

While we briefly describe probe design here, full probe design methodology, scripts and the probe set itself can be found at https://github.com/AAFC-BICoE/Elateridae-ortholog-baitset (accessed on 18 May 2021). Orthograph [27], in conjunction with the Coleoptera gene set from nine genomes available from OrthoDB v10 [28], was run on seven Elateroidea transcriptomes: *Monocrepidius* sp. (Elateridae: Agrypninae), *Drilus concolor* Ahrens, 1812 (Elateridae: Agrypninae), *Melanotus cribricollis* Candèze, 1860 (Elateridae: Elaterinae), *Melanotus villosus* (Geoffroy, 1785) (Elateridae: Elaterinae), *Photinus pyralis* (Linnaeus, 1763) (Lampyridae: Lampyrinae), *Chauliognathus flavipes* (Fabricius, 1781) (Cantharidae), and *Pyrearinus fragilis* Costa, 1978 (Elateridae: Agrypninae). NCBI repositories and respective accession numbers for reference datasets are provided in Supplementary File S2. Orthogroups were aligned using T-Coffee 11.0.8 [29] for amino acids, followed by Tranalign (EMBOSS 6.6.0) [30] for nucleotides. A custom python script used a sliding window approach to identify conserved regions in the amino acid alignments and excise the corresponding regions from the nucleotide alignments. To balance probe set size and cost, a final count of 2260 target regions, each representing a single-copy ortholog, were selected based on their representation in at least five of the eight Elateroidea reference taxa. These target regions were submitted as a multi-FASTA file to Arbor Biosciences (Ann Arbor, MI, USA) for the development of a myBaits custom probe kit. The final probe set contained 40,190 bait sequences of 100 nt lengths with staggered placement at 1.15X tiling. The probe set was first tested in silico with all 18 beetle genomes then available on NCBI (see GitHub link above) using the corresponding part of the Phyluce pipeline [31], after first converting FASTA headers to the format required by Phyluce. While in silico recovery using Phyluce was low (156–703 loci), only one genome tested was from series Elateriformia, and this genome (Buprestidae: *Agrilus planipennis* Fairmaire, 1888) was not from superfamily Elateroidea.

### 2.3. Sample Preparation and DNA Extraction

All vouchers were deposited at the Canadian National Collection of Insects, Arachnids and Nematodes (Ottawa, ON, Canada) and given identifiers as indicated in Supplementary Table S1. Most beetles were severed at the connection between prothorax and elytra. The whole bisected specimen or thoracic muscles (with, in some cases, a foreleg) were used for DNA extraction. For large specimens (>2 cm), only a foreleg was used. Before nondestructive DNA extraction, ethanol-preserved specimens were dried in a vacuum centrifuge to remove residual ethanol. Non-extracted parts of vouchers were stored at −20 °C in 95% ethanol, and extracted specimens were mostly card mounted dry after washing in ethanol. In all cases, signs of extraction were minor (slight colour lightening) or undetectable (most specimens). Specimens varied in age from 18 years to less than one year from the date of collection to the time of DNA extraction (2000–2017). DNA was extracted from alcohol-preserved specimens using a DNeasy™ blood and tissue kit (Qiagen, Montréal, Canada). In contrast, pinned specimens were extracted using a Qiagen QIAamp DNA micro kit (standard protocol with RNA carrier added). Four μL of RNase A (100 mg/mL) were added to each alcohol preserved sample to remove RNA, followed by a 2 min incubation at room temperature. In all cases, elution buffer was preheated to ~60 ℃ and DNA was eluted in buffer EB after a 10 min incubation. This step was repeated twice for a final elution volume of 30 μL. Three specimens were extracted using QuickGene DNA tissue kits DT-S (Kurabo, Osaka, Japan). Samples were treated with RNase A (20 uL at 100 mg/mL) before loading on the QuickGene 810 instrument set to “DNA TISSUE” mode. The elution time and volume parameters were set to 510 seconds and 50 μL of buffer CDT.

### 2.4. Library Preparation, Hybridization and Sequencing

Genomic DNA sample concentration was quantified using a Qubit 3.0 fluorometer dsDNA HS assay (Invitrogen, Waltham, MA, USA), and fragment quality was assessed using a 4200 TapeStation gDNA assay (Agilent Technologies, Santa Clara, CA, USA). DNA libraries were prepared using a NEBNext Ultra II FS kit for Illumina (New England BioLabs, Ipswich, MA, USA). DNA was sheared enzymatically to an average length of ~350 bp using incubation times of 1–15 min depending on each DNA sample’s initial average fragment size. Adaptors were diluted to 0.6 μM for DNA input <50 ng or 1.5 μM for DNA input between 50 and 100 ng. Adaptor ligated inserts were eluted in 33 μL 0.1X TE to increase insert recovery from SPRI beads. Libraries were dual-indexed using corresponding NEBNext multiplex oligos for Illumina (dual index primers set 1) and PCR-amplified for 7 or 9 cycles depending on the amount of input DNA for each sample (highest cycles for the lowest DNA concentrations). Library yield was quantified by Qubit fluorometer (Invitrogen).

Libraries were pooled at equal concentrations (50 ng per library) in groups of 10–13 per hybridization reaction, then reduced to ~7 μL using a vacuum centrifuge. Pooled libraries were exposed to a custom RNA probe kit (myBaits target capture kit Cat#300216, Ref#190812-91, Arbor Biosciences). Hybridization reactions were prepared according to the myBaits v.4.01 protocol, using 24-hour incubations at 65 °C and following the KAPA HiFi on bead PCR method. The purified, hybridized libraries were amplified with 16 cycles of PCR and cleaned with 0.8× SPRI beads. To determine the molarity and overall quality for sequencing, target enriched sample concentrations were assessed by Qubit, average fragment size was determined by 4200 TapeStation high-sensitivity D1000 assay. Sequencing viability was verified by qPCR (KAPA library quantification kit) on a Roche LightCycler 480. Equimolar pooled enriched libraries were sequenced at the Molecular Technologies Laboratory (Agriculture and Agri-Food Canada, Ottawa, ON, Canada) in multiple runs (~50 samples each) on an Illumina MiSeq using 600-cycle v3 kits. Demultiplexed, raw read FASTq files were deposited in the NCBI SRA under BioProject PRJNA717687.

### 2.5. Read Assembly and Orthology Assessment Pipeline

A bioinformatics pipeline, heavily drawing upon elements of the Phyluce package [31], was developed using Snakemake [32] to input raw Illumina reads and output aligned target loci for various target enrichment projects. This pipeline is available at https://github.com/AAFC-BICoE/snakemake-partial-genome-pipeline (accessed on 19 May 2021). Briefly, raw reads were first adapter-trimmed using BBDuk [33]. Single reads were then assembled de novo using three different assemblers: Abyss [34], g [35], and rnaSPAdes [36]. Deduplication and quality trimming were not performed because of the short length of the DNA fragments analyzed from specimens of variable preservation quality. Reads were merged using BBMerge and then assembled via a second run of Abyss. The output from each of the four assembly methods and the probe sequences were input separately into Phyluce, where assemblies were matched to target loci with a minimum 80% identity and 82% minimum coverage (defaults) to exclude contaminants. Assemblies matching multiple target genes were filtered out with Phyluce. Target genes with probes matching to multiple assemblies were treated as different and removed. These four assembly methods were compared, and the longest fragment for each target locus was retained. We have found that using multiple assemblers drastically increased the number of recovered targets, in agreement with the results of Hedin et al. [37].

### 2.6. Alignment, Trimming and Manual Inspection

Alignment and internal trimming were performed using elements of the Phyluce pipeline under default settings [31] unless otherwise stated. Alignment of each gene was performed in MAFFT [38] with edge trimming turned off. Internal trimming of ambiguously aligned regions was performed in Gblocks [39]. The trimmed, single-gene alignments were manually inspected in Geneious v10.2.6 (https://www.geneious.com, accessed on 20 August 2020) to find the reading frame, and address alignment artifacts, such as taxa with empty sequences (an artifact of earlier Gblocks step), and to remove taxa with very short sequences (<9 bp, after Phyluce and Gblocks trimming) as a result of trimming. The remaining misaligned and contaminant sequences not already filtered by Phyluce were identified by their broad disagreement with the amino acid level consensus and were removed.

Non-coding flanking regions were identified in Geneious as regions demarcated by stop codons, extreme deviations from the amino acid consensus and gaps not divisible by 3. Flanking regions were excised from multiple sequence alignments and included downstream (see below) in various analyses combined with probe regions. Probe regions were trimmed to start with codon position one, and misaligned gaps in coding probe regions and downstream nucleotides affected by the frameshift were converted to ambiguous (N’s).

### 2.7. Phylogenetic Analyses

Each gene alignment was placed into one or both of two sets, with at least 50 and 75 percent of taxa present, using the Phyluce script “phyluce_align_get_only_loci_with_min_taxa” [31]. Data from each set were concatenated into 50% and 75% probe alignments and 50% and 75% flanking alignments using AMAS [40]. Analyses were performed at the nucleotide (e.g., 50CP = 50% completeness matrix, concatenated, partitioned, nucleotide analysis) or amino acid level (e.g., 75CU-AA = 75% completeness matrix, concatenated, unpartitioned, amino acid analysis). Concatenated nucleotide datasets either included (e.g., 50CUF) or excluded (50CU) the flanking regions and were analyzed as partitioned (50CP, 75CP) and unpartitioned (50CU, 75CU) data, using maximum-likelihood (ML) in IQ-TREE v1.6 [41]. Amino acid datasets did not contain non-coding flanking regions.

The concatenated multiple sequence alignments were initially partitioned by codon position per gene to determine the optimal partitioning scheme using Bayesian information criterion (BIC) (PartitionFinder 2, [42]. Flanking regions, where included (e.g., 50CPF), were considered a single, entire candidate partition. Branch lengths were set to “linked”, and the search was set to use the relaxed clustering algorithm (rcluster) [43] in RAxML [44], with only the top 10% of schemes examined. Models were restricted to variants of GTR to reduce computational burden following Espeland et al. [45] and Gough et al. [46]. Merged partitions were then submitted to IQ-TREE, where the model selection was performed with all models considered (-m TESTNEW). Extremely small final partitions (<80 bp) returned by PartitionFinder 2, which typically contained only one gene position, were excluded because these caused the IQ-TREE analysis to fail at various points. These trimmed partitions represented only 1046 bp of 177,803 bp.

Partitioned analyses in IQ-TREE were performed with the -spp option following Duchêne et al. [47], and clade support was assessed using 1000 iterations of both the ultrafast bootstrap (UFB) [48] and an SH-aLRT test (SHT) [49]; the -nni option was used to avoid overestimation of bootstrap support in the presence of a violation of model assumptions [41]. Additional analyses of the CP50 dataset were conducted in IQ-TREE as above, but with third codon positions omitted from probe regions (e.g., 50CP-no3) to examine the potential effects of saturation on tree reconstruction and to resolve potential supported topological disagreements between nucleotide and amino acid datasets.

Coalescent analyses were performed in ASTRAL III v.X [50] on both the nucleotide and amino acid datasets (e.g., Astral50-AA, Astral50-n). Individual gene trees were generated using IQ-TREE; the substitution model was selected by BIC using ModelFinder (-m MFP). Near-zero branch lengths were collapsed using the “--polytomy” option. The latter collapses clades with extremely low support values (<10 UFB), which can cause errors in the reconstruction of the species tree in ASTRAL [50]. Analyses in ASTRAL were run with default parameters, and clade supports were calculated as the local posterior probability (LPP). All analyses were run either on the NCR-HPC-Biocluster at Agriculture and Agri-Food Canada (Ottawa, Canada) or the CIPRES Science Gateway v3.3 [51]. We considered UFB values ≥ 0.95, SHT values ≥ 80, or LPP ≥ 0.85 to indicate moderate support. Nodes with support from both UFB and SHT ≥ 0.95 or from LPP ≥ 0.95 were considered strongly supported. Nodes with support from only UFB or SHT, or LPP = 0.85–0.94 were considered weakly supported. Tree diagrams were visualized using a newly developed computing program (Supplementary File S3).

### 2.8. Four-cluster Likelihood Mapping

We used four-cluster likelihood mapping (FcLM) analyses [52,53,54] to investigate statistical support for alternative topologies recovered by our analyses, which may have been obscured by competing for phylogenetic signals. In FcLM, taxa are grouped into four-taxon sets representing a condensed topology around the node in question. These sets are assumed to be monophyletic. The graphical output of FcLM displays the proportion of taxon quartets that support each of the three possible topologies, are inconclusive between two alternatives, or are not supportive of any topology. FcLM analyses were performed in IQ-TREE 1.6, using -lmclust -lmap ALL and -n 0 options.

## 3. Results

### 3.1. Dataset and Target Capture

Of the 2260 targeted loci, 1536 were successfully recovered after Phyluce quality control filtering, with 300 to 1114 successfully recovered from each sample (Supplementary File S2). The probe set performed best within Elateridae (recovery: 537 to 1114 loci; average: 957 loci, *n* = 84), Phengodidae (753, *n* = 1), Lampyridae (729, *n* = 1), Cantharidae (700, *n* = 1), Rhagophthalmidae (689, *n* = 1), Artematopodidae (650, *n* = 1), Lycidae (576, *n* = 1), and least well in Eucnemidae (508, *n* = 1), Throscidae (337, *n* = 1), and Cerophytidae (300, *n* = 1). The two dried museum specimens yielded 576 (Lycidae) and 885 loci (Elateridae). Target recovery from published transcriptomes was high within Elateroidea (average: 960, four non-elaterid elateroids; 1285, 2 elaterids). After manual processing of single-locus alignments, the 50% and 75% completeness datasets contained 958 (177,803 bp) and 389 loci (100,948 bp), respectively. Flanking regions represented an additional 38,235 bp and 20,108 bp for 50% and 75% completeness sets, respectively. Concatenated datasets, according to the best scheme of PartitionFinder2, were divided into 110 (50CP) and 108 (75CP) final partitions (and are available here: https://doi.org/10.6084/m9.figshare.14619267.v1, accessed on 19 May 2021).

### 3.2. Phylogenetic Analyses

**Figure 2:**
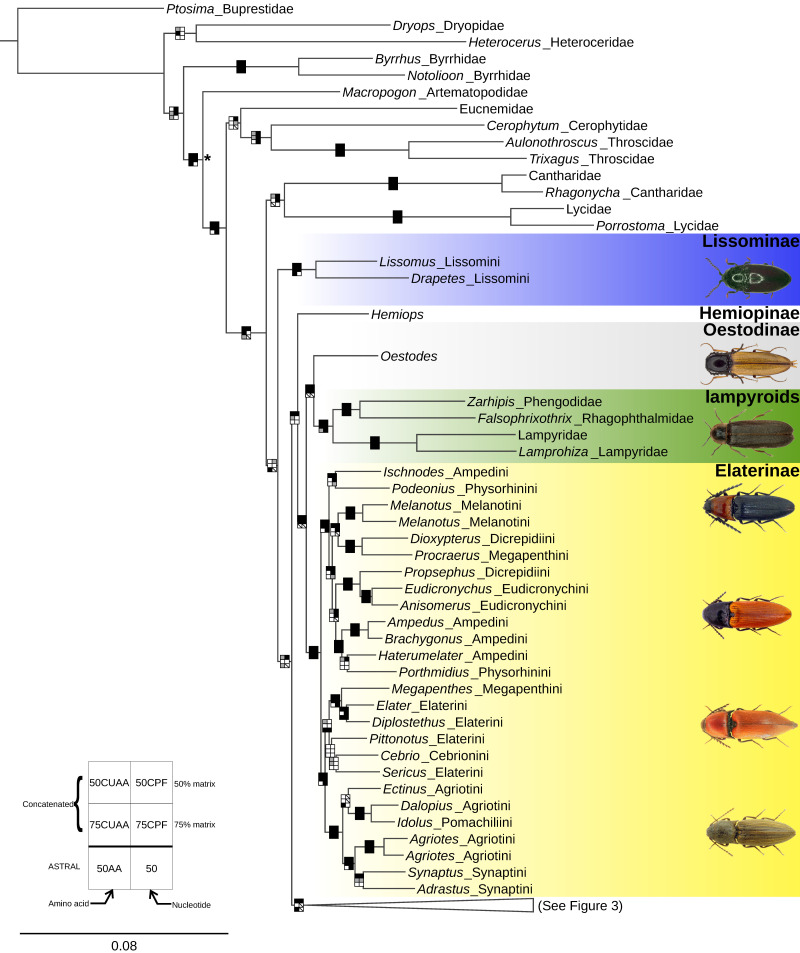
Phylogeny of the Elateroidea inferred from unpartitioned maximum-likelihood analysis of the 50% completeness amino acid matrix (50CUAA—958 loci). An asterisk (*) indicates the root of Elateroidea. Node boxes correspond to individual analyses, as shown in lower-left key and are shaded according to support: black—strong; gray—weak; white—unsupported; slash—strong support for an alternate topology. Beetle images from top to bottom: Lissominae (*Lissomus* sp.), Oestodinae (*Oestodes tenuicollis* (Randall, 1838)), Lampyridae (*Lampyris noctiluca* Linnaeus, 1767), Elaterinae (*Ischnodes sanguinicollis* (Panzer, 1793), *Ampedus sanguineus* (Linnaeus, 1758), *Elater ferrugineus* Linnaeus, 1758, *Agriotes lineatus* (Linnaeus, 1767)). Images of Elaterinae are from www.elateridae.com with permission (accessed on 8 January 2021).

**Figure 3:**
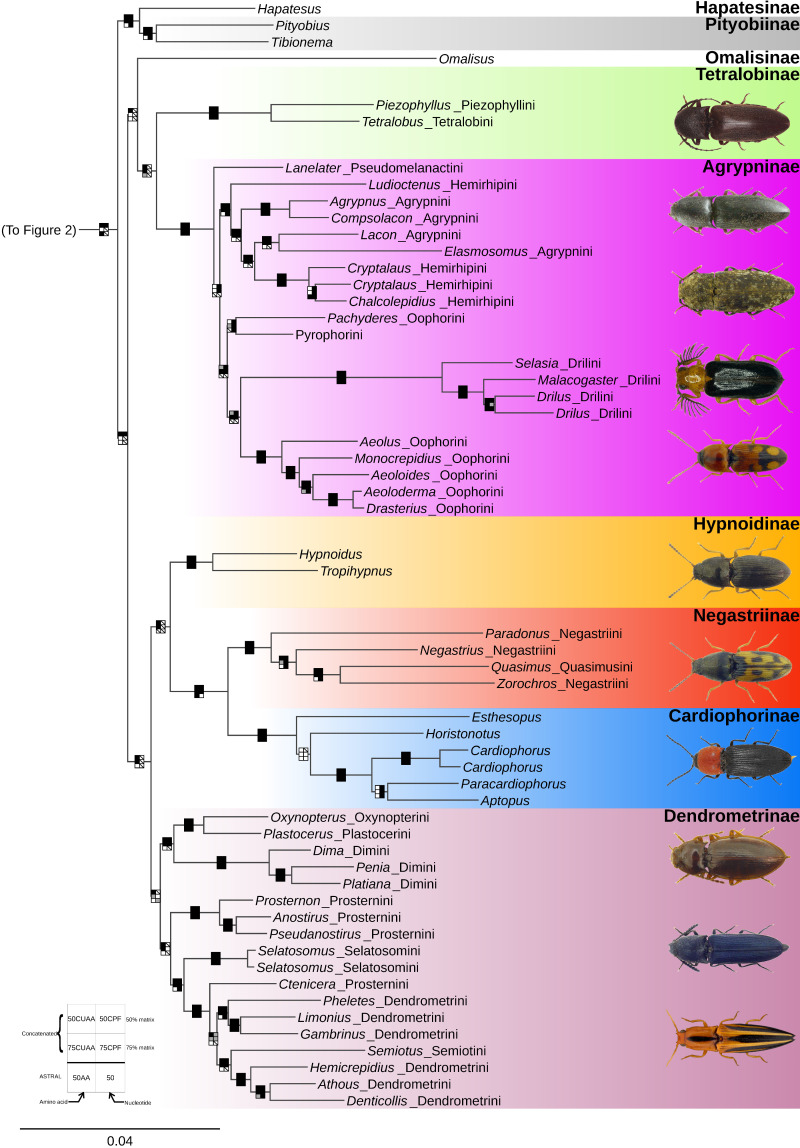
Phylogeny of the Elateroidea inferred from unpartitioned maximum-likelihood analysis of the 50% completeness amino acid matrix (50CUAA—958 loci). Node boxes correspond to individual analyses, as shown in the lower-left key and are shaded according to support: black—strong; gray—weak; white—unsupported; slash—strong support for an alternate topology. Beetle images from top to bottom: Tetralobinae (*Piezophyllus* sp.), Agrypninae (*Lanelater persicus* (Candèze, 1874), *Agrypnus murinus* (Linnaeus, 1758), *Selasia* sp., *Drasterius bimaculatus* (Rossi, 1790)); Hypnoidinae (*Hypnoidus consobrinus* (Mulsant and Guillebeau, 1855)), Negastriinae (*Negastrius sabulicola* (Boheman, 1854)), Cardiophorinae (*Cardiophorus gramineus* (Scopoli, 1763)), Dendrometrinae (*Dima elateroides* Charpentier, 1825, *Gambrinus violaceus* (Müller, 1821), *Semiotus furcatus* (Fabricius, 1792)). All images, except *Piezophyllus* sp., *Selasia* sp. and *Semiotus furcatus*, are from www.elateridae.com with permission (accessed on 8 January 2021).

Topologies generated by analyses of concatenated datasets, particularly of the 50% completeness matrices, were well-resolved and mostly congruent, with few conflicts between well-supported branches (Figure 2 and Figure 3, Table 1, Supplementary File S4). The results from the 75CPF dataset disagreed with those from the 50CPF dataset, resulting in differing placements of Tetralobinae, Cardiophorinae and Negastriinae. However, the placement of these taxa was highly influenced by third codon positions (below). The 75% completeness datasets are not discussed further because they were less informative than the 50% completeness datasets (Supplementary File S4). Similarly, analyses with flanking regions included differed little from those where they were excluded (Table 1) and are not discussed separately. Results from partitioned analyses were more resolved in nucleotide analyses and preferred for discussion, but not so for amino acids (Table 1). The conflicts from these topologies are discussed separately below in the context of subsequent analyses.

**Figure 4:**
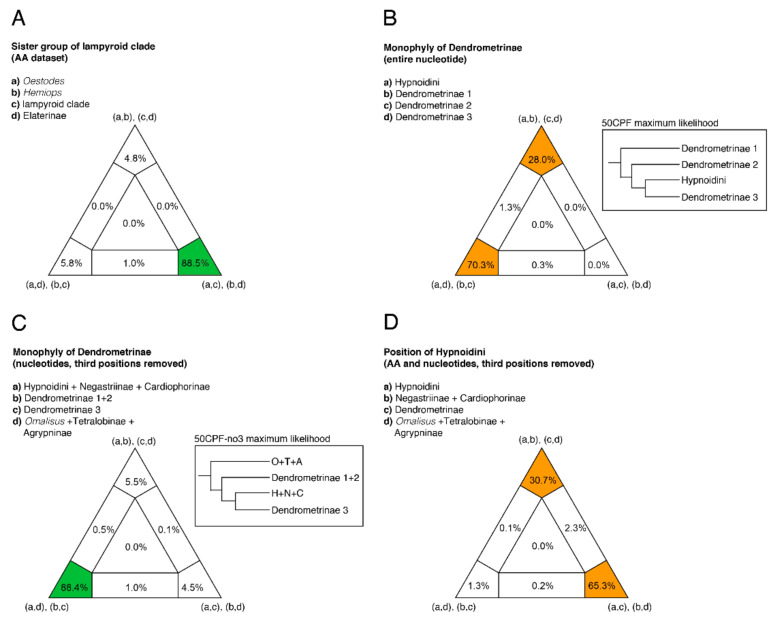
Four cluster likelihood mapping (FcLM) tests of alternative phylogenetic hypotheses: (**A**) the position of the lampyroid clade; (**B**) monophyly of Dendrometrinae, with taxon sets as resolved by concatenated analysis of entire nucleotide dataset (inset phylogeny); (**C**) monophyly of Dendrometrinae, with taxon sets as resolved by concatenated analysis of nucleotide dataset, third positions removed (inset phylogeny); (**D**) the position of Hypnoidini, monophyly of remaining Dendrometrinae assumed (results of AA dataset shown, nucleotide dataset with third positions removed nearly identical). Dendrometrinae 1 = *Prosternon* Latreille, 1834, *Anostirus* Thomson, 1859, and *Pseudanostirus* Dolin, 1964; Dendrometrinae 2 = *Oxynopterus* Hope, 1842, *Plastocerus*, and Dimini; Dendrometrinae 3 = *Selatosomus*, *Ctenicera*, *Semiotus*, and Dendrometrini; O + T + A = *Omalisus* + Tetralobinae + Agrypninae; H + N + C = Hypnoidini + Negastriinae + Cardiophorinae.

Lissominae were recovered as sister to lampyroids + the remaining Elateridae in all analyses, except the coalescent analysis of nucleotide data, where its position was unresolved. Concatenated amino acid and nucleotide datasets agreed that the lampyroids rendered the Elateridae paraphyletic in the following configuration ((lampyroids + *Oestodes* LeConte, 1853) + Elaterinae) + *Hemiops* Laporte, 1838 (Figure 1 and Figure 2, Table 1, Supplementary File S4). Coalescent analyses of nucleotide and amino acid data agreed on the placement of lampyroids within Elateridae, but as follows: lampyroids as sister to the clade *Hemiops* + (*Oestodes* + Elaterinae), or an unresolved clade of *Hemiops* + *Oestodes* + Elaterinae. Coalescent analysis of nucleotide data also found Cantharidae, Cerophytidae and Throscidae separately as part of Elateridae. These topologies were not suggested in other studies (or elsewhere here) and are not discussed further. FcLM analysis of the 50CUAA dataset recovered unambiguous support for the concatenated result (*Oestodes* + lampyroids) versus the coalescent result (*Oestodes* + Elaterinae) (Figure 4A). Despite some topological variation, the lampyroids were recovered inside Elateridae in all of our analyses.

The highest level of contradiction across analyses was between nucleotide and amino acid datasets. For example, Tetralobinae were sister to Agrypninae in all amino acid analyses, but sister to Cardiophorinae + Negastriinae in the nucleotide analyses. Additionally, Hypnoidini were sister to Cardiophorinae + Negastriinae in the concatenated amino acid analyses but nested alone within Dendrometrinae in the concatenated nucleotide analyses. In agreement with concatenated analyses of nucleotide data, all coalescent analyses recovered a clade consisting of Hypnoidini and Dendrometrinae. However, coalescent analyses recovered Hypnoidini as sister to the remaining Dendrometrinae. Reanalysis of concatenated nucleotide data with third codon positions removed (both unpartitioned and partitioned, and without and with flanking regions) resulted in well-supported topologies consistent with amino acid datasets (Table 1, Supplementary file S4, “no3” trees): Tetralobinae + Agrypninae; and Hypnoidini sister to Cardiophorinae + Negastriinae. Unlike the amino acid results, the latter clade was always nested inside a paraphyletic Dendrometrinae. However, this topology was supported only in the 50CP analysis.

To test the possibility that Cardiophorinae + Negastriinae + Hypnoidini cause paraphyly of the remaining Dendrometrinae, we conducted FcLM analyses on the two nucleotide datasets which produced this result. First, FcLM analysis showed that, while a clade formed by Hypnoidini + *Selatosomus* Stephens, 1830, *Ctenicera* Latreille, 1829. *Semiotus* Eschscholtz, 1829 and Dendrometrini (Figure 4B, inset phylogeny) was fully supported in analyses of the full nucleotide dataset, nearly one-third of the total phylogenetic signal conflicted (Figure 4B). Although nucleotide analyses with third positions removed recovered a paraphyletic Dendrometrinae (Figure 4C, inset phylogeny), an overwhelming majority of the phylogenetic signal within this dataset surprisingly supported its monophyly (minus Hypnoidini) (Figure 4C). Thus, saturated synonymous changes to third positions were considered responsible for the compositional bias signal for this node in the full nucleotide datasets (Figure 4B). With their removal, the lesser but substantial signal in support of monophyletic Dendrometrinae (minus Hypnoidini) (Figure 4B) became dominant (Figure 4C) and in agreement with our other analyses (concatenated AA, all coalescent analyses). Next, treating Dendrometrinae as a monophyletic taxon set, we conducted FcLM analyses on both amino acid and nucleotide (no third positions) datasets to assess support for conflicting concatenated and coalescent topologies regarding the position of Hypnoidini. FcLM analyses (datasets within 0.5% of each other) found approximately two-thirds of the phylogenetic signal in support of Hypnoidini sister to Dendrometrinae (coalescent result). In contrast, almost one-third of the signal supported Hypnoidini sister to the clade Negastriinae + Cardiophorinae (concatenated result) (Figure 4D).

Beyond the above three conflicts, there was substantial agreement between all analyses above and below the family level (Table 1). Most notable among these are the nested position of the monophyletic lampyroids within Elateridae and the monophyly of Pityobiinae (including *Tibionema*) plus Hapatesinae. All analyses recovered *Plastocerus* Schaum, 1852 inside Dendrometrinae, as sister to Oxynopterini, and Semiotini were recovered within tribe Dendrometrini. Here Dimini were monophyletic, but Prosternini, including Selatosomini, were paraphyletic.

## 4. Discussion

Our novel, elateroid-focused AHE probe set successfully enriched as many as 1114 loci per specimen and recovered many well-supported Elateroidea clades. Gene recovery was successful from both ethanol preserved and dried specimens, as reported by Brunke et al. [55] and Shin et al. [24]. Between the two dried specimens, the lycid specimen had a lower number of genes recovered despite more recent collection into 95% ethanol, so that genes recovered reflect perhaps the relative affinity of the probes to the target taxon more than the preservation method of the specimens. Target recovery was highest in Elateridae, including the lampyroid clade. Recovery was lower in the elaterid subfamilies Cardiophorinae and Negastriinae and lowest in families Eucnemidae, Throscidae, and Cerophytidae. The lower recovery rates for specimens of the last three families may have contributed to lower branch support levels for their placement in the amino acid trees. Integration of transcriptome data (*Byrrhus pilula*, *Drilus concolor*, *Dryops* sp., *Heterocerus fenestratus*, *Lamprohiza splendidula*, *Melanotus villosus*, *Notolioon* sp., *Porrostoma* sp., *Ptosima undecimmaculata*, *Rhagonycha fulva*, *Trixagus carinifrons*) into our dataset demonstrates the forward compatibility of data from this bait set with such data.

Using the resulting dataset for phylogenetic analysis, sister group relationships within Elateroidea and Elateridae were generally well resolved, and node support was highest when more loci were included in the analyses despite increased missing data rates. The finding that results from 50% completeness matrices were more robust than those from 75% matrices is consistent with simulation findings by Molloy and Warnow [56] that filtering to remove loci with greater proportions of missing data can result in loss of useful phylogenetic signal.

Although our tree recovered tribe and subfamily groupings consistent with previous DNA-based phylogenies, we report new findings in areas of the phylogeny that were formerly poorly resolved, as well as conflicts between our nucleotide and amino acid data. Here, amino acid trees agreed more with Douglas’ [9] morphological hypotheses than did nucleotide trees. Nucleotide trees with third-codon positions excluded agreed with amino acid trees for all well-supported but conflicting nodes, providing consistent results. This suggests that conflicting signals from saturated third positions obscured phylogenetic signals and generated a topological error. Our analyses reveal new insights into the phylogeny of the Elateridae and suggest that the lampyroids (Lampyridae, Phengodidae and Rhagophthalmidae) may need to be incorporated within the Elateridae.

### 4.1. Monophyly of the Elateridae

Our study indicates that the lampyroids render Elateridae paraphyletic (Figure 2, Table 1). This finding is consistent with all well-supported clades in previous molecular studies and is also suggested by several of these studies [2,3,6]. This finding requires a detailed reexamination of elaterid and lampyroid morphology, chemical defenses, and biology to understand the evolutionary transitions implied by our results. Such reconsideration is not new for Elateridae. For example, softer bodied or paedomorphic Cebrionidae, Drilidae, and Omalisidae were all recently demonstrated to be part of Elateridae [10,16]. However, prior detailed comparisons of larval morphology by Beutel [57] found shared unique traits between lampyroids and members of the cantharoid clade (Cantharidae + Lycidae), but not between the lampyroids and Elateridae. Similarly, we know of only one apparent synapomorphy of Elateridae (including the putative family Sinopyrophoridae), Lampyridae, Phengodidae, and Rhagophthalmidae: that these are the only extant families of Elateriformia that include bioluminescent species. The recovery of the lampyroid clade within Elateridae requires us to revisit whether bioluminescence evolved independently in Elateridae and lampyroids (e.g., [58]). Thylacosterninae, relatives of Lissominae [11,12,15], which were here sister to all other Elateridae + lampyroids, include bioluminescent species [59], (although see 58). This means that the earliest diverging subclade of Elateridae + the lampyroids also includes bioluminescent species. This suggests that the ancestor of Elateridae and the lampyroids was probably either bioluminescent or somehow preadapted for bioluminescence (e.g., possessing the necessary biochemical pathways). The finding that Lampyroids are derived Elateridae can inform future studies focused on the evolution of bioluminescence in this clade using research strategies like those of Oba et al. [60].

Elateridae includes both beneficial predators and notorious plant pests [7,61]. The recent incorporation of Drilidae into Elateridae demonstrated that the elaterid clade includes another group of predators of agricultural pests (especially the harmful and elsewhere invasive European land snails [62]). Here the demonstration that the lampyroids, and particularly Lampyridae, are also derived elaterids reveals many beneficial members of the elaterid clade (Elateridae, including the lampyroids). Lampyrid larvae have long been known to prey on various agricultural pests, especially gastropods [7,63,64]. Findings by Traugott et al. [65] indicate that even species presumed herbivorous pests may be predators. Together these findings indicate that the Elateridae include many species beneficial to agriculture and at least a pest species. These results also indicate that more research is needed to understand the roles of the many elaterid clades that are abundant in agricultural lands.

Although evidence that lampyroids are derived elaterids may appear novel, this agrees with published DNA-sequence-based analyses, despite limited taxon sampling. The results of Kusy et al. [6] that *Sinopyrophorus* is sister to Lampyridae or all lampyroids, and of Bi et al. [15] that *Sinopyrophorus* + *Hemiops* are sister to *Oestodes* are both here corroborated. These three well-supported inferences about sister group relationships suggest that *Sinopyrophorus*, *Oestodes*, *Hemiops*, Elaterinae and lampyroids may form a clade within Elateridae and that current evidence does not support a separate. Such recognition of Sinopyrophoridae would require more extensive taxon sampling and consensus on the taxonomic status of well-established families Elateridae, Lampyridae, Phengodidae, and Rhagophthalmidae. However, the inclusion of the three existing lampyroid families as valid at the family rank within Elateridae would require division of Elateridae into at least five families. Any further development of the classification of Elateroidea requires complementary morphological research to define synapomorphies and morphological diagnoses. The systematics of Elateroidea is already challenging, and the proposal of new families without an adequate diagnosis, e.g., Sinopyrophoridae [6], further entangle its taxonomy.

**Figure 5:**
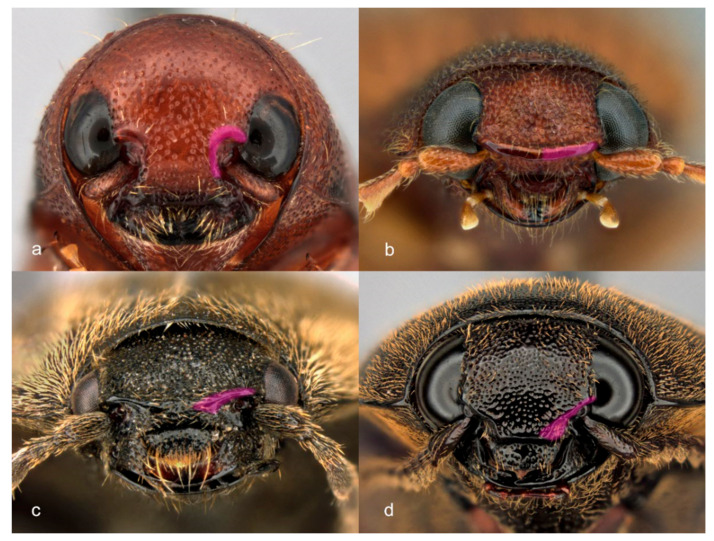
Frontal views of Elateridae heads showing character states of supra-antennal carinae with carina highlighted in part. (**a**) following outlines of antennal cavities (Thylacosterninae: *Pterotarsus bimaculatus* Laporte, 1835); (**b**) directed mesad and fused at midline forming shelf (Dendrometrinae: *Athous orvus* Becker, 1974); (**c**) directed mesad and fading near the midline (Dendrometrinae: *Prosternon* sp.); (**d**) directed anteromesad to the anterior edge of frontoclypeus (Elaterinae: *Orthostethus infuscatus* Germar, 1844).

Within Elateridae (as currently classified), subfamily Lissominae were sister to Elateridae, including the lampyroids, in most of our trees. This was with weak support in most trees and strong support in the coalescent analysis of amino acid data. Members of this group share adult external morphological similarities with the Eucnemidae [8], which were in the next most closely related clade of hard-bodied Elateroidea outside the Elateridae. The Eucnemidae are like Elateridae in their elongate shape and clicking ability. The elongate, cylindrical-bodied Thylacosterninae, in particular, has long been confused with those of Eucnemidae, both which have adaptations to development within cylindrical tunnels in wood. In addition to this, Lissominae share the eucnemid-type frons (e.g., *sensu* Fleutiaux [66]). Here the supra-antennal carinae follow the outline of the antennal fossae (ending near the mandible base) (Figure 5a), unlike other Elateridae where the supra-antennal carinae are directed mesad forming a shelf-like projection (Figure 5b), disappearing toward the midline (Figure 5c), or reaching the base of the labrum (Figure 5d) [9]. Hence, in addition to clicking ability, a cylindrical body shape and eucnemid-type frons are shared members of at least two distant elateroid families.

It is becoming evident that many soft-bodied Elateroidea evolved separately from hard-bodied, clicking ancestors. Here, it seems likely that Cantharidae and Lycidae (cantharoids) also evolved from hard-bodied, clicking ancestors. Hence, early stem cantharoids were perhaps also hard-bodied and likely shared the eucnemid-type frons because this frons type is present in both distantly related elateroids and in early-diverging lissomine and thylacosternine elaterids. Knowledge of this likely transition means that hard-bodied fossil elateroids may belong to hypothetical extinct hard-bodied cantharoid groups or to extant groups that have since softened. This possibility should promote caution in assigning hard-bodied fossil elateroids to any modern family (e.g., [67]) without a more detailed association via reexamined synapomorphies. Specifically, fossils resembling modern Eucnemidae may actually belong to Elateridae or to hard-bodied ancestral cantharoids or their morphologically similar, extinct relatives.

### 4.2. Major Divisions of the Elateridae and Their Morphology

Both our nucleotide and amino acid datasets found strong support for existing subfamily level clades of Elateridae as defined by morphological studies of adults and larvae, e.g., [68,69,70], and which were also recovered in previous phylogenetic analyses [12,15], including Agrypninae, Tetralobinae, Lissominae, Pityobiinae, Elaterinae, Cardiophorinae, and Negastriinae (Table 1). The present study also provides novel results at family and subfamily levels in areas where previous studies had insufficient inference power, i.e., few characters or few taxa sampled.

The monophyly of Elaterinae and several of its lineages were well supported. Our trees indicate that eventual taxonomic changes are required for the non-monophyletic tribes Agriotini, Ampedini, Dicrepidiini, Elaterini, Megapenthini and Physorhinini. Our results support treating Eudicronychini as a tribe of Elaterinae and suggest that they may be derived members of Dicrepidiini. Subfamily Elaterinae continues to be most often recognized by its hypognathous heads as adults but is more robustly diagnosed by well-sclerotized larvae without a caudal notch in abdominal tergite IX [1]. In all analyses, Elaterinae were composed of two main, robustly supported clades: the “*Elater* clade”, including members of Agriotini (non-monophyletic), Elaterini (non-monophyletic), Pomachiliini, Cebrionini, Synaptini, and *Megapenthes tartareus* (LeConte, 1859) (Megapenthini); and the “*Ampedus* clade”, including Ampedini (non-monophyletic), Dicrepidiini (non-monophyletic, as defined by Johnson [71]), Melanotini, Physorhinini (non-monophyletic), Eudicronychini, and *Procraerus* Reitter, 1905 (Megapenthini). Genera with the supra-antennal carinae (or together with the frontal carina) incomplete across the head (Figure 5d) were all resolved within the “*Elater* clade”, and all genera with lobed tarsi occurred in the “*Ampedus* clade”. Other characters, including complete supra-antennal carinae (Figure 5b); non-lobed tarsi; simple claws, and pectinate claws, occur in both clades. Although several tribes (see above) were found non-monophyletic, and their limits and diagnoses will need reexamination, only members of one tribe, Megapenthini, were found in both the *Elater* and *Ampedus* clades.

The “pityobiine clade” containing Hapatesinae (*Hapatesus*) and Pityobiinae (*Pityobius*, *Tibionema*) was found in all analyses (Table 1), which agrees with the mitogenomic analyses by Kusy et al. [19], who found *Hapatesus* sister to *Tibionema* (Pityobiinae) and *Parablax* Schwarz, 1906 (Parablacinae). Kundrata et al. [12] tested the monophyly of Pityobiinae, which at that time contained North American *Pityobius*, South American *Tibionema*, and several Australasian genera near *Parablax*. They found *Pityobius* unrelated to the Australasian genera, which they considered a separate subfamily Parablacinae, and kept *Tibionema* only tentatively in Pityobiinae. Here, *Tibionema* is confirmed to be closely related to *Pityobius*, as also found by Dolin [72], based on wing venation. Although Kusy et al. [19] found Pityobiinae related to Parablacinae, a clearer understanding of relationships within this early-diverging clade of Elateridae requires future analyses, including as many Australasian lineages as possible, and thorough reexamination of the morphology of Hapatesinae, Pityobiinae and Parablacinae. Some other similar Southern-Hemisphere taxa should be examined for evidence of possible membership in this clade. For example, the Australian *Rousia* Calder, 1996 possibly belongs to Parablacinae as it shares with them a similar frontoclypeal region, dorsally convex scutellar shield with anterior portion gradually elevated and without sharp carina, tarsomeres ventrally with spongiose pads, wing venation with two apical field sclerotizations at an acute angle to each other, and similar shape and arrangement of spines of the female bursa copulatrix [73], (R.K. personal observation). All well-supported trees (Table 1) found the pityobiine clade was monophyletic and sister to the remaining elaterid subfamilies together: the agrypnine clade (introduced below), Cardiophorinae, Negastriinae, Dendrometrinae: Hypnoidini, and the remaining Dendrometrinae (including Plastocerini).

Our finding of an “agrypnine clade”, where *Omalisus* is sister to Agrypninae + Tetralobinae (Table 1), is consistent with recognizing Omalisinae as valid at the subfamily level by Kusy et al. [16]. The alternate hypothesis supported by some analyses here that *Omalisus* is sister to Agrypninae without Tetralobinae also supports this validity. However, this alternate topology, where Tetralobinae was sister to Cardiophorinae + Negastriinae, was found only in the concatenated analysis of nucleotide data with often saturated third codon positions included. More kinds of evidence support placement of Tetralobinae as sister to Agrypninae, including all analyses of amino acid data and the nucleotide data with third codon positions deleted. In addition to strong evidence from the present phylogenomic analyses, we find this result convincing because Tetralobinae were viewed as sister to, or part of, Agrypninae based on adult and larval morphology [70] and phylogenetic analysis of adult morphology [9] and because the same trees are also congruent with the placement of Hypnoidini based on morphological evidence (see below). Conversely, no morphological synapomorphies uniting Cardiophorinae + Negastriinae with Tetralobinae have been proposed to date. We recommend no changes to the rank of Omalisinae because apomorphic morphological characters have not been found to unite it with Agrypninae or Tetralobinae. Further, we prefer to keep Tetralobinae as a separate subfamily from Agrypninae, pending a detailed analysis of Tetralobinae and Agrypninae. Our analyses also show that this agrypnine clade is sister to Cardiophorinae + Negastriinae + Dendrometrinae: Hypnoidini + the remaining Dendrometrinae (including Plastocerini).

Agrypninae, as currently defined [74], were consistently recovered here as monophyletic. The agrypnine tribe Pseudomelanactini, represented here by *Lanelater* Arnett, 1952, was sister to all other included genera in most analyses but possesses no known unique morphological synapomorphies. The Pseudomelanactini, with only two genera and about 100 species, are distributed in tropical and temperate regions worldwide. Of the remaining Agrypninae, one clade, including reciprocally paraphyletic Agrypnini and Hemirhipini, included mainly species where adults have thick exoskeletons and scale-like setae covering the body. The remaining genera included only thin-exoskeleton species with simple setae, including Pyrophorini, *Pachyderes* Guérin-Méneville, 1829, Drilini, and Oophorini. This second group includes most bioluminescent Agrypninae species, highly derived Drilini snail predators, and relatively homogeneous Oophorini, with tarsomere IV lobed and elytral striae not converging before the elytral apex. Most members of this group have supra-antennal carinae continuing shelf-like across the frons without interruption. However, these carinae are vague to absent in the soft-bodied Drilini [75].

The tribal classification of Agrypninae will need detailed revision in future studies. Although soft-bodied Drilini were always recovered here as monophyletic, Agrypnini, Hemirhipini, and Oophorini were not. Based on the clade formed here by *Lacon* and *Elasmosomus*, away from other Agrypnini, it may be necessary to consider the possible redefinition of Laconini [76] and its resurrection from synonymy under Agrypnini. In corroboration with our results, Hemirhipini was also non-monophyletic by molecular phylogenetic analyses [12,20] and larval morphology [77]. Consequently, it may be necessary to recognize *Chalcolepidius* Eschscholtz, 1829; *Cryptalaus* Ôhira, 1967; *Alaus* Eschscholtz, 1829 and relatives as tribe Chalcolepidiini Candèze, 1857, and distinct from Hemirhipini (Figure 3, Supplementary File S4), as found by Rosa et al. [77]. Most genera of Oophorini (i.e., *Aeolus* Eschscholtz, 1829, *Aeoloides* Schwarz, 1906, *Monocrepidius* Eschscholtz, 1829, *Aeoloderma* Fleutiaux, 1928, and *Drasterius* Eschscholtz, 1829) formed a fully supported clade in all analyses. However, genus *Pachyderes*, although placed there by some, e.g., [78], does not belong to a monophyletic Oophorini according to our results (Figure 3, Table 1). This finding suggests that future studies should consider the possible redefinition of Pachyderini Fleutiaux, 1919 and its resurrection from synonymy under Oophorini.

Hypnoidini were given a subfamilial rank by Stibick [70]), but a tribal rank within Dendrometrinae by Dolin [72]. Most recent authors considered hypnoidines as part of Dendrometrinae, based on shared wing venation characters [72]. This morphological evidence is congruent with the results of our coalescent analyses, where Dendrometrinae were supported as the sister group of Hypnoidini. It is also congruent with concatenated analyses of the full nucleotide dataset, where Hypnoidini were deeply nested within Dendrometrinae. Still, this result was possibly an artifact because support was largely confined to third positions (Figure 4B,C). However, with the removal of third positions, our concatenated nucleotide results agreed with those from the concatenated amino acid data in full support for the topology Hypnoidini + (Cardiophorinae + Negastriinae), consistent with the morphological phylogenetic results of Douglas [9]. In the analyses with third codon positions omitted, the Hypnoidini + Cardiophorinae + Negastriinae clade was nested within Dendrometrinae. However, FcLM analysis (Figure 4C) of this dataset strongly suggests that this placement was an artifact and demonstrates that the great majority of phylogenetic signal agrees with our other analyses in recovering Dendrometrinae (minus Hypnoidini) as monophyletic.

After an exploration of phylogenetic signal across various types of data and analytical methods, two supported phylogenetic hypotheses regarding the sister group of Hypnoidini remained: either sister to Negastriinae + Cardiophorinae (concatenated) or sister to Dendrometrinae (coalescent). FcLM analysis of both nucleotide and amino acid datasets showed substantial conflict in the phylogenetic signal, with the coalescent hypothesis receiving slightly more than twice as much support (Figure 4D). We conclude that our phylogenomic data alone are currently unable to resolve this conflict. However, there is considerable morphological evidence in support of the concatenated hypothesis.

All members of the hypothesized clade Hypnoidini + (Cardiophorinae + Negastriinae) share possible nonunique synapomorphies of a complete supra-antennal shelf and partially or completely closed mesocoxal cavities. Douglas [9] also found that the hypnoidine *Tropihypnus* Reitter, 1905 shared unique prosternal and head synapomorphies with Negastriinae and Negastriinae + Cardiophorinae, respectively, suggesting possible paraphyly of hypnoidines. The supra-antennal carina is bifurcate near both eyes only in Hypnoidini, Negastriinae and some Cardiophorinae, representing an apparent unique synapomorphy for the three. Additionally, the aedeagi of Cardiophorinae, Negastriinae and some Hypnoidini have apparently unique parameres that are basally fused or constrained as a tube by membranes, unlike any Dendrometrinae. Larval Negastriinae and Hypnoidini share an apical notch in abdominal tergite IX and simple urogomphi. This is an additional apparent synapomorphy for these two since the urogomphi are bifid in most Dendrometrinae. The mostly soft-bodied larvae of Cardiophorinae have non-sclerotized abdomens so that any urogomphi or notches would be structurally unsupported, and so perhaps developmentally impossible. Further possible autecological evidence uniting Hypnoidini, Negastriinae and Cardiophorinae is that many Hypnoidini, most Negastriinae, and some Cardiophorinae inhabit riparian and littoral zones, with adults often burrowing into loose substrates. This habitat and behavior are infrequently associated with any other clade. Here, adult *Rismethus scobinula* Candèze, 1857 in Agrypnini similarly inhabit riparian gravel (H.B.D. personal observation), although they are not known to share these morphological synapomorphies as adults [79] or larvae ([69], from congeneric species).

All of these character systems and microhabitat preferences provide evidence that is consistent with a single lineage of Hypnoidini, Cardiophorinae, and Negastriinae, as indicated by our concatenated results. This hypothesis is incompatible with a tribal rank for hypnoidines, and therefore, we treat the lineage as Hypnoidinae **stat. rev**. In the context of the alternative, coalescent hypothesis, where hypnoidines are sister to the rest of Dendrometrinae, separate subfamily status remains preferred to improve the morphological diagnosis of diverse Dendrometrinae.

The Dendrometrinae, including Plastocerini, but not Hypnoidinae, when recovered as monophyletic, were composed of two major clades, one, including *Plastocerus* + Oxynopterini + Dimini, and the other Dendrometrini + Semiotini + Prosternini + Selatosomini. The monophyly of such widely delimited Dendrometrinae is supported by all amino acid analyses and coalescent analysis of nucleotide data but contradicted by other analyses of nucleotide data. The inclusion of *Plastocerus* within Dendrometrinae agrees with Bocak et al. [14] and Kusy et al. [16], indicating that the Plastocerini should be treated as a tribe of widely delimited Dendrometrinae. All trees also show Prosternini and Dendrometrini as non-monophyletic as currently defined, e.g., [78,80,81]. This means that further phylogenetic analysis is needed to determine the status of these tribes, perhaps supporting the recognition of additional tribes beyond the recently named Selatosomini Schimmel et al. 2015. This would most likely include Ctenicerini Jakobson, 1913 for *Ctenicera* and relatives, and Denticollini Stein and Weise, 1877 for *Denticollis* Piller and Mitterpacher, 1783, *Athous*, *Hemicrepidius* Germar, 1839 and relatives. *Semiotus* is deeply nested in the current Dendrometrini in all supported results of nucleotide and amino acid analyses, indicating the status of the enigmatic New World Semiotini should be further explored with increased taxon sampling and morphological examination.

Subfamily Dendrometrinae is most often recognized by adults with flattened, hypognathous heads and by larvae with a caudal notch in abdominal tergite IX. Within the Dendrometrinae, members of the clade (Oxynopterini + Plastocerini) + Dimini (each found monophyletic) all share prognathous mouthparts and an absence of complete supra-antennal carinae across the frons. The other clade contains members of Prosternini, Selatosomini, Dendrometrini, and Semiotini, all of which (except Semiotini) are characterized by larvae having a rectangular or broadly rounded submentum. In our analyses, the Selatosomini plus the paraphyletic Prosternini formed a grade of taxa recognized as adults by their simple tarsi and supra antennal carinae fading medially (Figure 5c) and not forming a continuous shelf. Dendrometrini + Semiotini formed a terminal clade with joined supra-antennal carinae forming a shelf across the head (Figure 5b) and with some species also having lobed tarsomeres.

While the present study allows major new insights into the evolution of Elateridae, further knowledge could be obtained by adding sequences from the remaining elaterid subfamilies. It is particularly important to obtain data for Campyloxeninae, Parablacinae, Subprotelaterinae, *Sinopyrophorus*, and Thylacosterninae. Obtaining data from Morostominae and Physodactylinae would also be useful. However, available evidence suggests these might be examples of Dendrometrinae and Elaterinae, respectively [15]. We also recommend adding data from southern hemisphere genera currently assigned to Dendrometrinae, and Hemiopinae, as initiated by Kundrata et al. [12]. These subfamily placements were often made based on habitus-level similarities (e.g., hypognathous vs. prognathous mouthparts), which should not be treated as strong evidence for membership in any subfamily.

## 5. Conclusions

We report the successful development of a novel AHE probe set for Elateroidea. Analyses of the resulting data showed a strong phylogenetic signal and demonstrated forward compatibility with transcriptomic data. In the first application of this probe set, we found that Lampyridae, Phengodidae, and Rhagophthalmidae form a clade within a paraphyletic Elateridae, as sister to *Oestodes*. Hence, we found that lampyroids are modified elaterid click-beetles, so that the entire clade contains over 13,500 species. While this finding implies that major taxonomic changes are needed to Elateridae or to all these families (plus fossil Cretophengodidae [82]), we argue that such formal incorporation of lampyroids should await further study. We do not recommend changes to the rank of Sinopyrophoridae. However, our results demonstrate that recognition at family rank is currently unjustified. We generally urge our colleagues to wait for supporting evidence before making taxonomic changes. Waiting is particularly justified now as new phylogenetic evidence is expected to continue providing answers about these and other long-standing questions. This finding implies yet another independent origin of soft-bodiedness within Elateridae, implying that hard-bodied fossils resembling Eucnemidae or Elateridae may be more closely related to currently soft-bodied groups.

The monophyly of elaterid subfamily Dendrometrinae excluding *Plastocerus* was rejected. However, the monophyly each of Elaterinae, Pityobiinae, Agrypninae, Tetralobinae, Hypnoidinae, Cardiophorinae and Negastriinae was consistent. We propose the following changes, Hypnoidinae **revised status** and Plastocerini **new status** in Dendrometrinae instead of a subfamily. Many tribal level groups were found to be non-monophyletic here. Most notable among these are that Semiotini fall within currently defined Dendrometrini. Eudichronychini are also confirmed to be most closely related to some Dicrepidiini. We hope that this study will be useful as a foundation for future phylogenomic studies to address persistent questions about the subfamily and tribal classification and the position of lampyroids within a now larger Elateridae.

## Figures and Tables

**Table 1:** Support for clades in analyses of 50% completeness matrices. Support format is UFB/SHT for concatenated analyses and LPP alone for coalescent analyses (two rightmost columns). Results indicating strongly supported clades are bolded. Clades that include members of multiple families are highlighted blue, and clades with members of multiple elaterid subfamilies are in orange. Strengths and weaknesses of individual analyses are treated in the discussion. C = concatenated; AS = ASTRAL (coalescent); U = unpartitioned; P = partitioned; F = with flanking regions added; no3 = third codon positions omitted; n = nucleotide data; aa= amino acid data; NR = clade not recovered. Dendrometrinae* = incl. Oxynopterini, *Plastocerus*, Dimini, Dendrometrini, Semiotini, Prosternini, and Selatosomini.

Clades/Analyses	CU aa	CP aa	CU n	CP n	CPF n	CPF no3	CU no3	CP no3	AS aa	AS n
Lissominae + (Elateridae + lampyroids)	79/71	89/75	89/91	85/82	91/89	83/79	67/39	77/67	**0.95**	NR
Elateridae	NR	NR	NR	NR	NR	NR	NR	NR	NR	NR
Lissominae	**100/100**	**100/100**	**100/100**	**100/100**	**100/100**	**100/100**	**100/100**	**100/100**	**1**	NR
Elateridae + lampyroids (minus Lissominae)	**100/93**	**100/96**	**100/100**	**100/100**	**100/100**	**100/100**	**100/97**	**100/99**	**0.87**	NR
*Oestodes* + lampyroids	**98/98**	**99/97**	**100/100**	**100/100**	**100/100**	**100/100**	**100/100**	**100/100**	NR	NR
*Oestodes* + lampyroids + Elaterinae	**100/100**	**100/100**	**100/100**	**100/100**	**100/100**	**100/100**	**100/100**	**100/100**	NR	NR
*Oestodes* + lampyroids + *Hemiops* + Elaterinae	**100/100**	**100/100**	**99/99**	**100/100**	**100/100**	**100/100**	**100/100**	**100/100**	0.68	0.81
Elaterinae (incl. Cebrionini, Aplastini, Eudicronychini)	**100/100**	**100/100**	**100/100**	**100/100**	**100/100**	**100/100**	**100/100**	**100/100**	**1**	**1**
Elaterinae: “*Ampedus* clade”	**100/100**	**100/100**	**100/100**	**100/100**	**100/100**	**100/100**	**100/100**	**100/99**	0.71	**1**
Elaterinae: “*Elater* clade”	**100/100**	**100/99**	**100/100**	**100/100**	**100/100**	**100/100**	**100/100**	**100/100**	NR	**1**
Each of Agriotini; Ampedini; Dicrepidiini; Elaterini; Megapenthini; and Physorhinini	NR	NR	NR	NR	NR	NR	NR	NR	NR	NR
Each of Eudicronychini and Synaptini + Agriotini + Pomachiliini	**100/100**	**100/100**	**100/100**	**100/100**	**100/100**	**100/100**	**100/100**	**100/100**	**1**	**1**
*Pityobius* + *Tibionema* (=Pityobiinae)	**99/99**	**100/99**	**100/100**	**100/100**	**100/100**	**100/100**	**100/100**	**100/100**	0.67	**1**
*Pityobius* + *Tibionema* + *Hapatesus*	**100/100**	**100/100**	**100/100**	**100/100**	**100/100**	**100/100**	**100/100**	**100/100**	0.84	**1**
*Pityobius* + *Tibionema* + *Hapatesus* + *Omalisus* + Agrypninae + Tetralobinae + Cardiophorinae + Negastriinae + Hypnoidini + Dendrometrinae*	**100/100**	**100/99**	NR	**100/99**	**100/100**	**100/100**	**100/99**	**100/100**	**0.99**	NR
*Omalisus* + Agrypninae + Tetralobinae + Cardiophorinae + Negastriinae + Hypnoidini + Dendrometrinae*	**100/96**	100/93	NR	**100/99**	**100/100**	**100/100**	**100/99**	**100/100**	0.49	NR
*Omalisus* + Agrypninae + Tetralobinae	**97/95**	96/90	NR	NR	NR	**100/100**	**100/99**	**100/100**	0.54	NR
Agrypninae + Tetralobinae	**100/100**	**100/100**	NR	NR	NR	96/85	87/82	**99/96**	**0.91**	NR
*Omalisus* + Agrypninae	NR	NR	**100/100**	**100/100**	**100/100**	NR	NR	NR	NR	NR
Each of Agrypninae and Tetrolobinae	**100/100**	**100/100**	**100/100**	**100/100**	**100/100**	**100/100**	**100/100**	**100/100**	**1**	**1**
Each of Agrypnini (incl. *Lacon* Laporte, 1838 and *Elasmosomus* Schwarz, 1902); and Hemirhipini (incl. *Ludioctenus* Fairmaire, 1893); and Oophorini	NR	NR	NR	NR	NR	NR	NR	NR	NR	NR
Each of Oophorini (excl. *Pachyderes* Guérin-Méneville, 1829) and Drilini	**100/100**	**100/100**	**100/100**	**100/100**	**100/100**	**100/100**	**100/100**	**100/100**	**1**	**1**
Cardiophorinae + Negastriinae + Hypnoidini + Dendrometrinae *	**100/100**	**100/100**	NR	NR	NR	**100/100**	**100/100**	**100/100**	0.49	NR
Cardiophorinae + Negastriinae + Hypnoidini	**100/100**	**100/100**	NR	NR	NR	**100/100**	**100/100**	**100/100**	NR	NR
Cardiophorinae + Negastriinae + Tetralobinae	NR	NR	**100/100**	**100/100**	**100/100**	NR	NR	NR	NR	NR
Cardiophorinae + Negastriinae	**100/100**	**100/100**	**100/100**	**100/100**	**100/100**	**100/100**	**100/100**	**100/100**	**1**	NR
Each of Cardiophorinae and Hypnoidini	**100/100**	**100/100**	**100/100**	**100/100**	**100/100**	**100/100**	**100/100**	**100/100**	**1**	**1**
Negastriinae	**100/100**	**100/100**	**100/100**	**100/100**	**100/100**	**100/100**	**100/100**	**100/100**	**0.99**	**1**
Hypnoidini + Dendrometrinae*	NR	NR	**100/ 100**	**100/ 100**	**100/ 100**	NR	NR	NR	**1**	**1**
Dendrometrinae* (without Hypnoidini)	**98/96**	**99/97**	NR	NR	NR	NR	NR	NR	0.67	**0.92**
Dendrometrinae (without *Plastocerus*)	NR	NR	NR	NR	NR	NR	NR	NR	NR	NR
Oxynopterini + *Plastocerus* + Dimini	**100/100**	**100/100**	**100/100**	**100/100**	**100/100**	**100/100**	**100/100**	**100/100**	NR	NR
Each of Oxynopterini + *Plastocerus* and Dimini	**100/100**	**100/100**	**100/100**	**100/100**	**100/100**	**100/100**	**100/100**	**100/100**	**1**	**1**
Dendrometrini + Semiotini + Prosternini + Selatosomini + Hypnoidini	NR	NR	**100/100**	**100/100**	**100/100**	NR	NR	NR	**1**	NR
Prosternini + Selatosomini + Ctenicerini	NR	NR	NR	NR	NR	NR	NR	NR	NR	NR
Dendrometrini excl. Semiotini	NR	NR	NR	NR	NR	NR	NR	NR	0.39	NR
Dendrometrini incl. Semiotini	**99/99**	**100/99**	94/90	94/89	89/87	96/87	96/93	**99/96**	NR	NR
Each of Denticollina + *Athous* Eschscholtz, 1829 + Hemicrepidiina and Dendrometrina (minus *Athous*)	**100/100**	**100/100**	**100/100**	**100/100**	**100/100**	**100/100**	**100/100**	**100/100**	0.79	**1**
Denticollina + *Athous* + Hemicrepidiina + Semiotini	**100/100**	**100/100**	**100/100**	**100/100**	**100/100**	**100/100**	**100/100**	**100/100**	NR	NR

## Data Availability

The data that support the findings of this study are openly available in the GitHub repositories at: https://github.com/AAFC-BICoE/elateridae-ortholog-baitset (accessed on 19 May 2021), and https://github.com/AAFC-BICoE/snakemake-partial-genome-pipeline (accessed on 19 May 2021).

## Supplementary Materials

The following are available online at https://www.mdpi.com/article/10.3390/biology10060451/s1, Supplementary Table S1: Specimens included in trees, Supplementary File S2: Genetic resources used for Phyluce quality control filtering, Supplementary file S3 Pseudocode for development of computer software to generate support matrices for Figure 2 and Figure 3, Supplementary File S4: Tree files.
