# Supplementary material for: Anchored Phylogenomics, Evolution and Systematics of Elateridae: Are All Bioluminescent Elateroidea Derived Click Beetles?
Source: Biology (Basel). 2021 May 21;10(6):451. doi: 10.3390/biology10060451 (PMC8224040; doi:10.3390/biology10060451)
Supplement: Supplementary file 1 [file biology-10-00451-s001.zip › Douglas_Suppl_S3_Pseudocode.pdf]

```
‖ Function to return cell shading of branch n_a (in preferred tree t_a), given topology of alternate tree t_b
function colour_cell (node n_a, tree t_b) -> colour
        let s_a = set of all terminals apical to n_a
        let n_b = most recent common ancestor of all s_a in t_b
        let s_b = set of all terminals apical to n_b
        if (size of s_a == size of s_b) then :
                    ‖ Nodes are equivalent in both trees
              return colour corresponding to confidence of n_b in t_b
        else :
              let t_x = collapse_tree(n_b)
              let s_x = s_b - s_a
              for each child i of t_x :
                     let s_i = set of all terminals apical to i
                     if (s_i is a subset of s_x) then :
                            remove i from t_x
                            let s_x = s_x – s_i
                     end if
              end for each
              if (size of s_x > 0) then :
                            ‖ Could not split extra terminals under node n_b into their own branch
                     return colour corresponding to strongly-supported alternate topology
              end if
              for each child i of n_a :
                     let s_i = set of all terminals apical to i
                     for each child j of t_x :
                            let s_j = set of all terminals apical to j
                            if (s_j is a subset of s_i) then :
                                   remove j from t_x
                                   let s_i = s_i – s_j
                            end if
                     end for each
                     if (size of s_i > 0) then :
                                   ‖ Could not separate terminals of branch under n_a from collapsed sub-tree under n_b
                            return colour corresponding to strongly-supported alternate topology
                     end if
              end for each
                    ‖ Equivalent node was recovered after splitting off extra terminals
              return colour corresponding to weakly-supported node
        end if
end function

‖ Function to return an equivalent tree to a sub-tree starting from node n with all nodes with weak confidence collapsed (removed)
function collapse_tree(node n) -> tree
        let result = empty tree
        if (confidence of n is strong) then :
              for each child i of n :
                     add collapse_tree(i) as child of result
              end for each
        else :
              for each child i of n :
                     let t_x = collapse_tree(i)
                     for each child j of t_x :
                            add j as child of result
                     end for each
              end for each
        end if
        return result
end function
```

Supplementary File S3 Pseudocode presenting the algorithm used to determine the branch support cell shading at nodes in figures 2 and 3
