# Supplementary material for: Anchored Phylogenomics, Evolution and Systematics of Elateridae: Are All Bioluminescent Elateroidea Derived Click Beetles?
Source: Biology (Basel). 2021 May 21;10(6):451. doi: 10.3390/biology10060451 (PMC8224040; doi:10.3390/biology10060451)
Supplement: Supplementary file 1 [file biology-10-00451-s001.zip › Douglas_Suppl_S4_Trees.pdf]

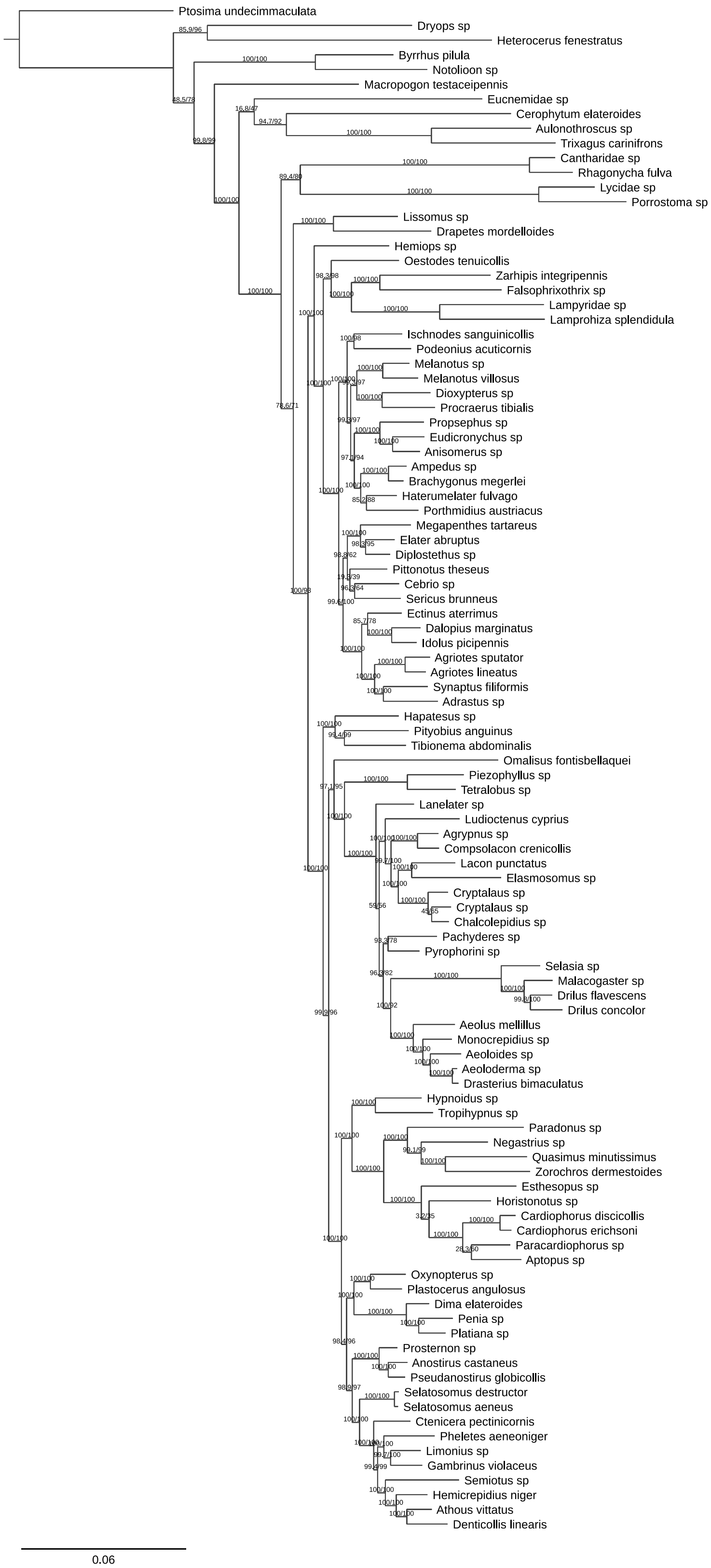

Phylogeny inferred from maximum likelihood analysis of unpartitioned, concatenated amino acid data, 50% completeness matrix (50CU-AA)

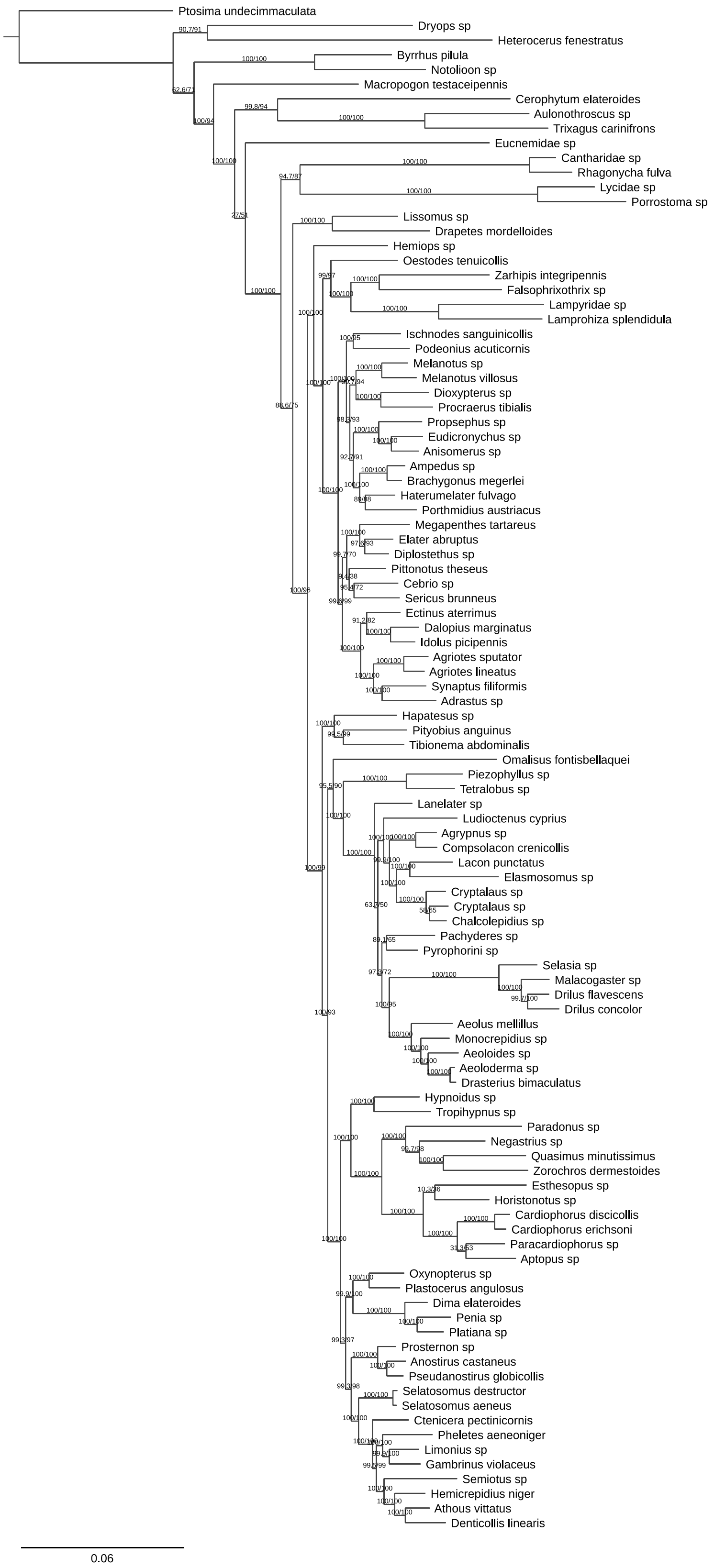

Phylogeny inferred from maximum likelihood analysis of partitioned, concatenated amino acid data, 50% completeness matrix (50CP-AA)

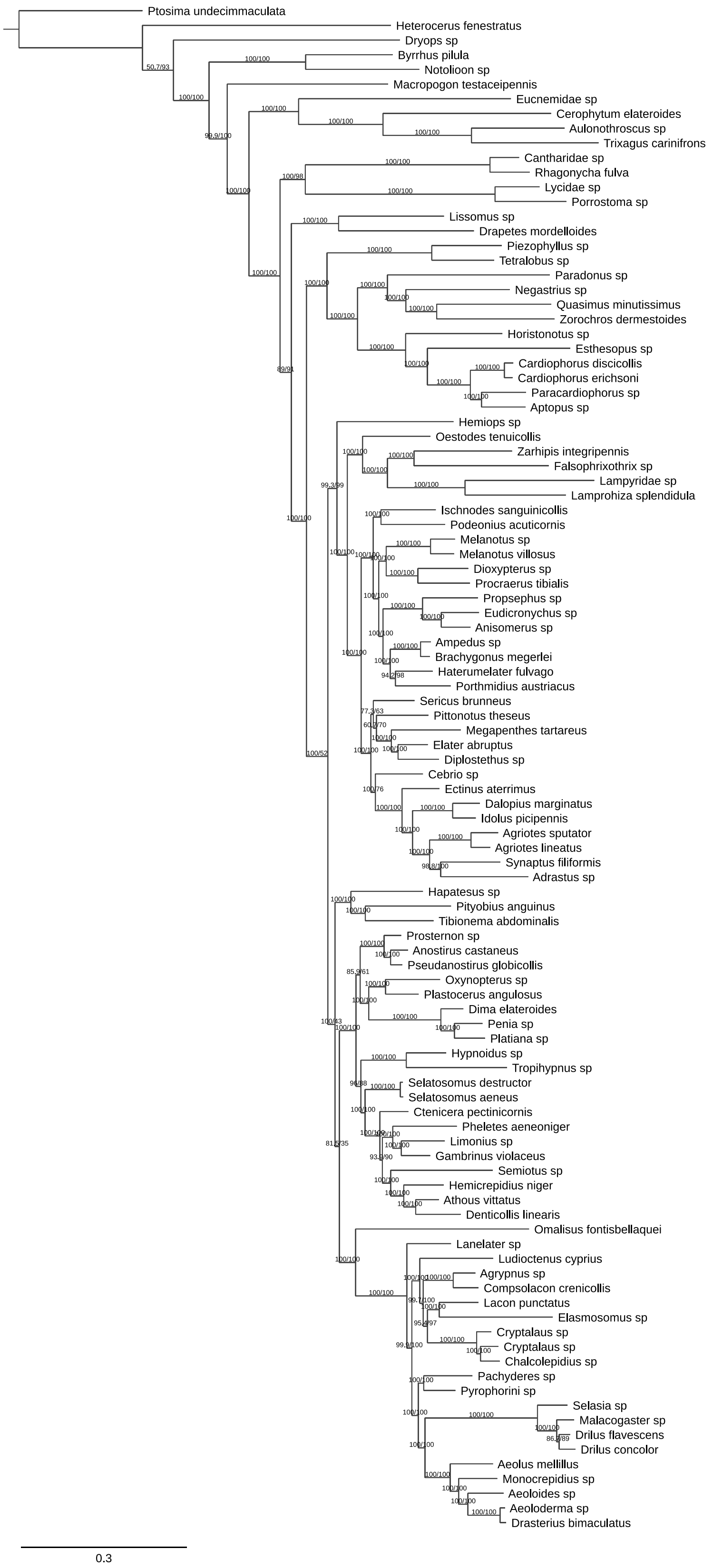

Phylogeny inferred from maximum likelihood analysis of unpartitioned, concatenated nucleotide data, 50% completeness matrix (50CU)

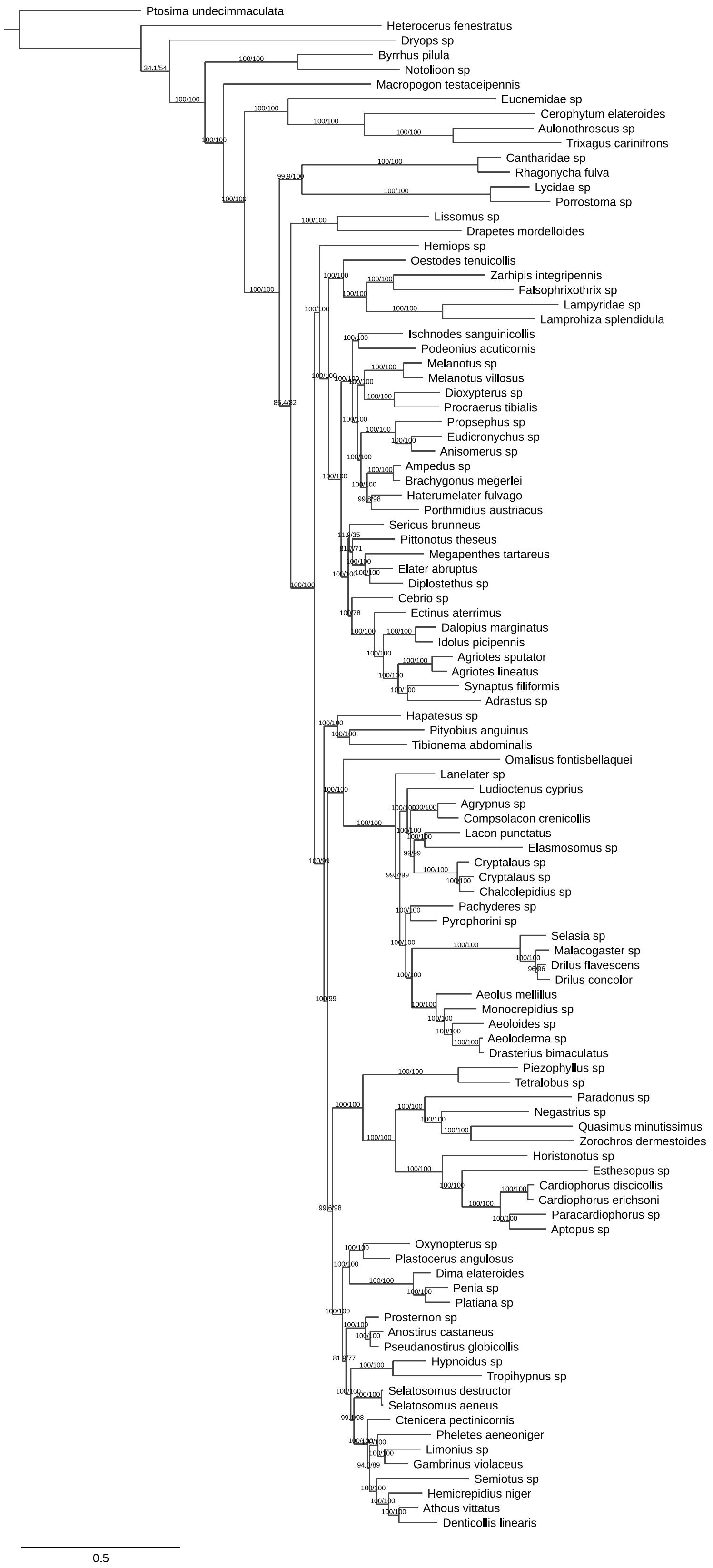

Phylogeny inferred from maximum likelihood analysis of partitioned, concatenated nucleotide data, 50% completeness matrix (50CP)

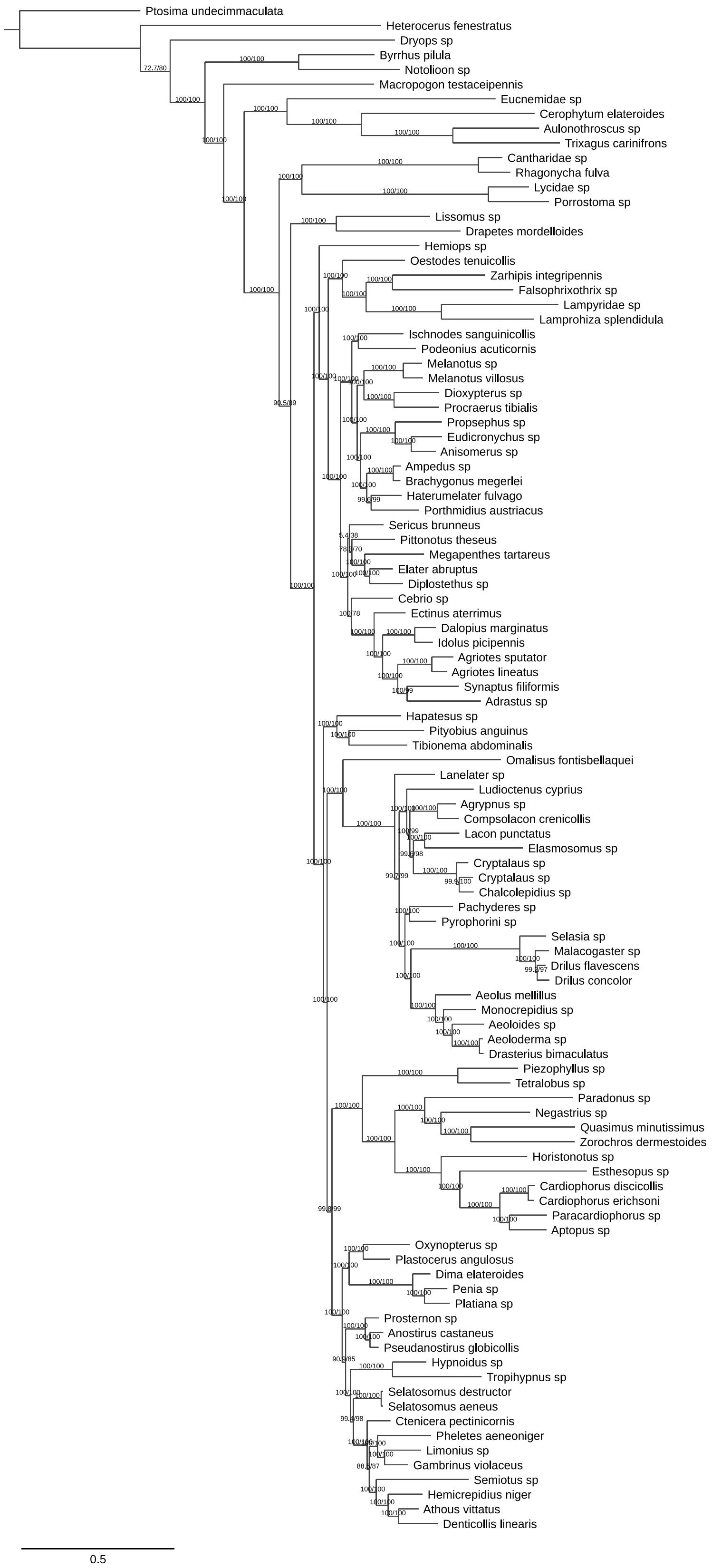

Phylogeny inferred from maximum likelihood analysis of partitioned, concatenated nucleotide data with flanking regions added, 50% completeness matrix (50CPF)

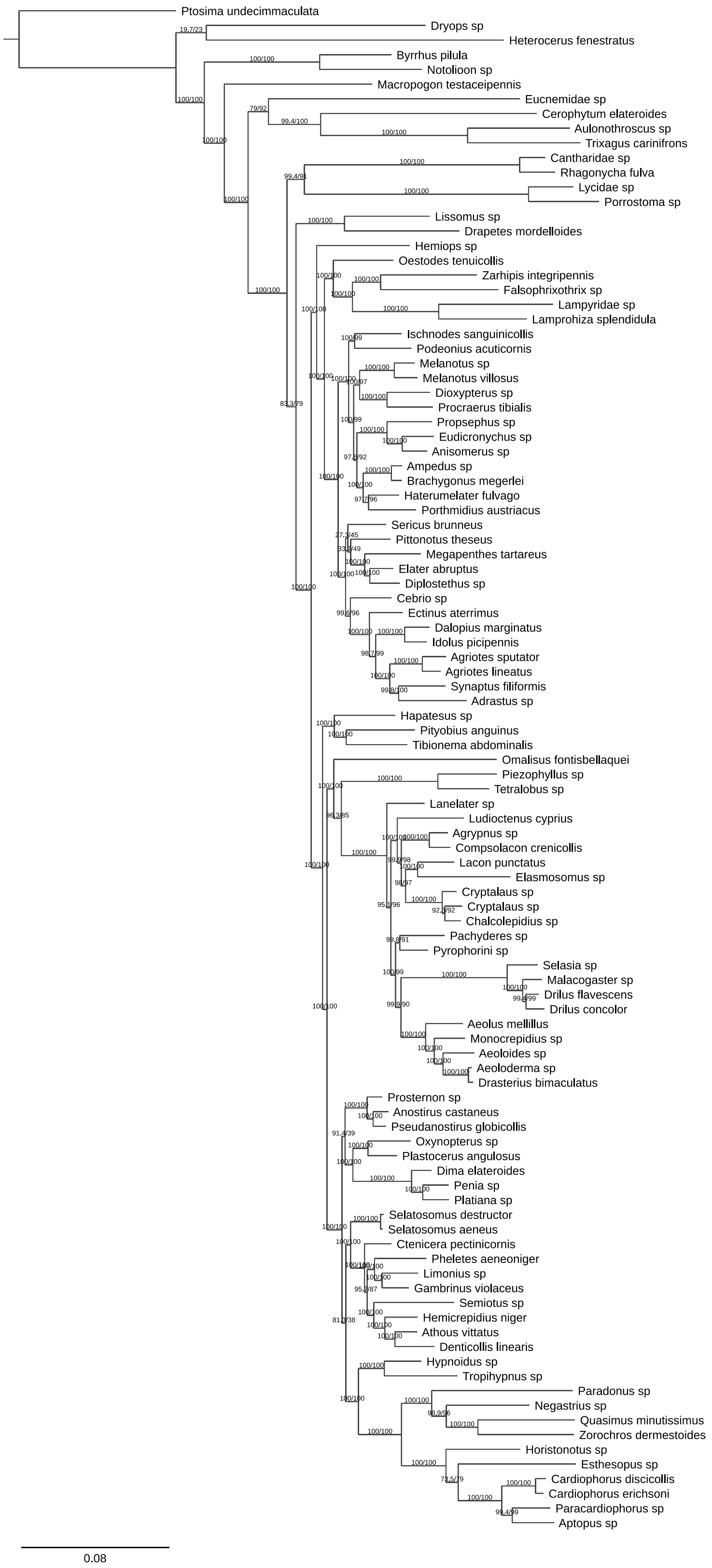

Phylogeny inferred from maximum likelihood analysis of partitioned, concatenated nucleotide data with flanking regions added and third codon positions removed, 50% completeness matrix (50CPF-No3)

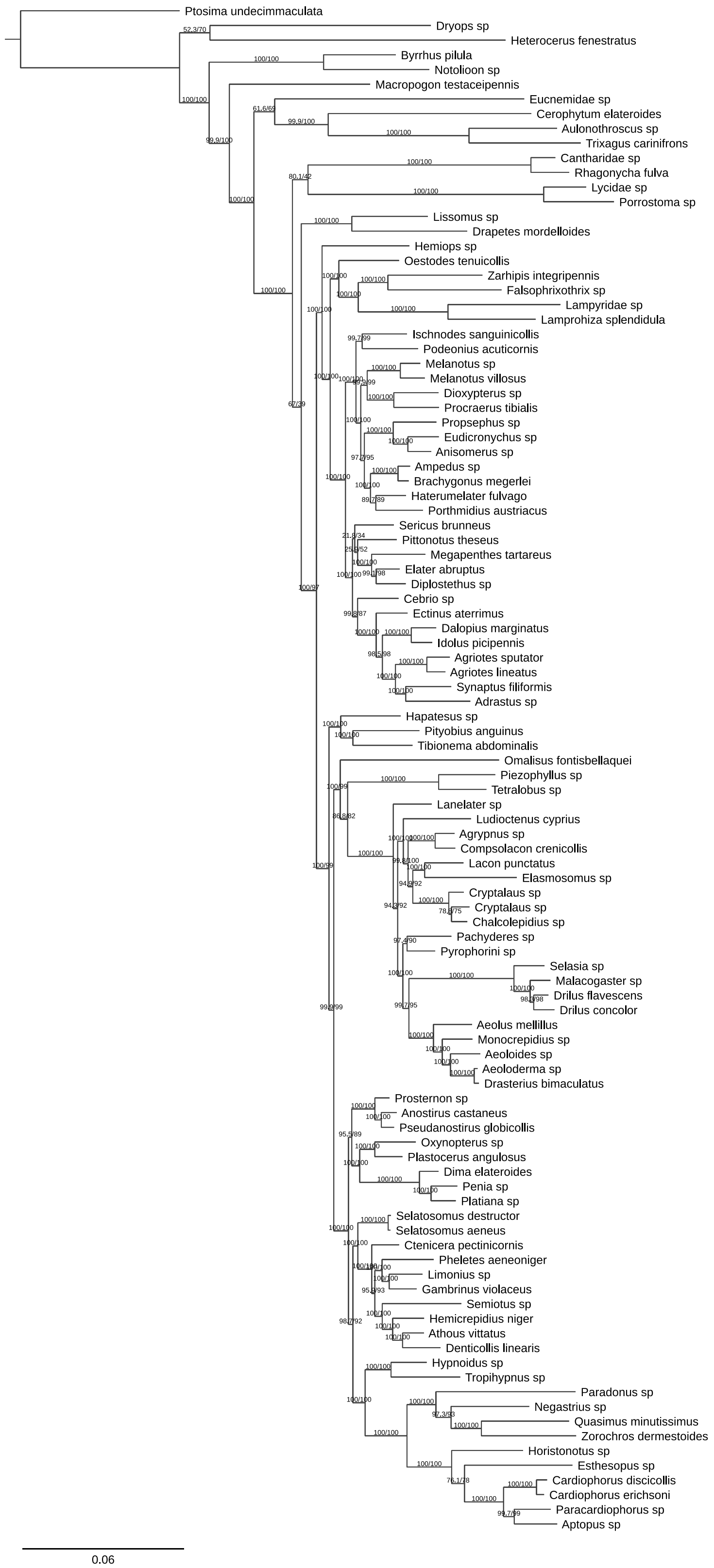

Phylogeny inferred from maximum likelihood analysis of unpartitioned, concatenated nucleotide data with third codon positions removed, 50% completeness matrix (50CU-No3)

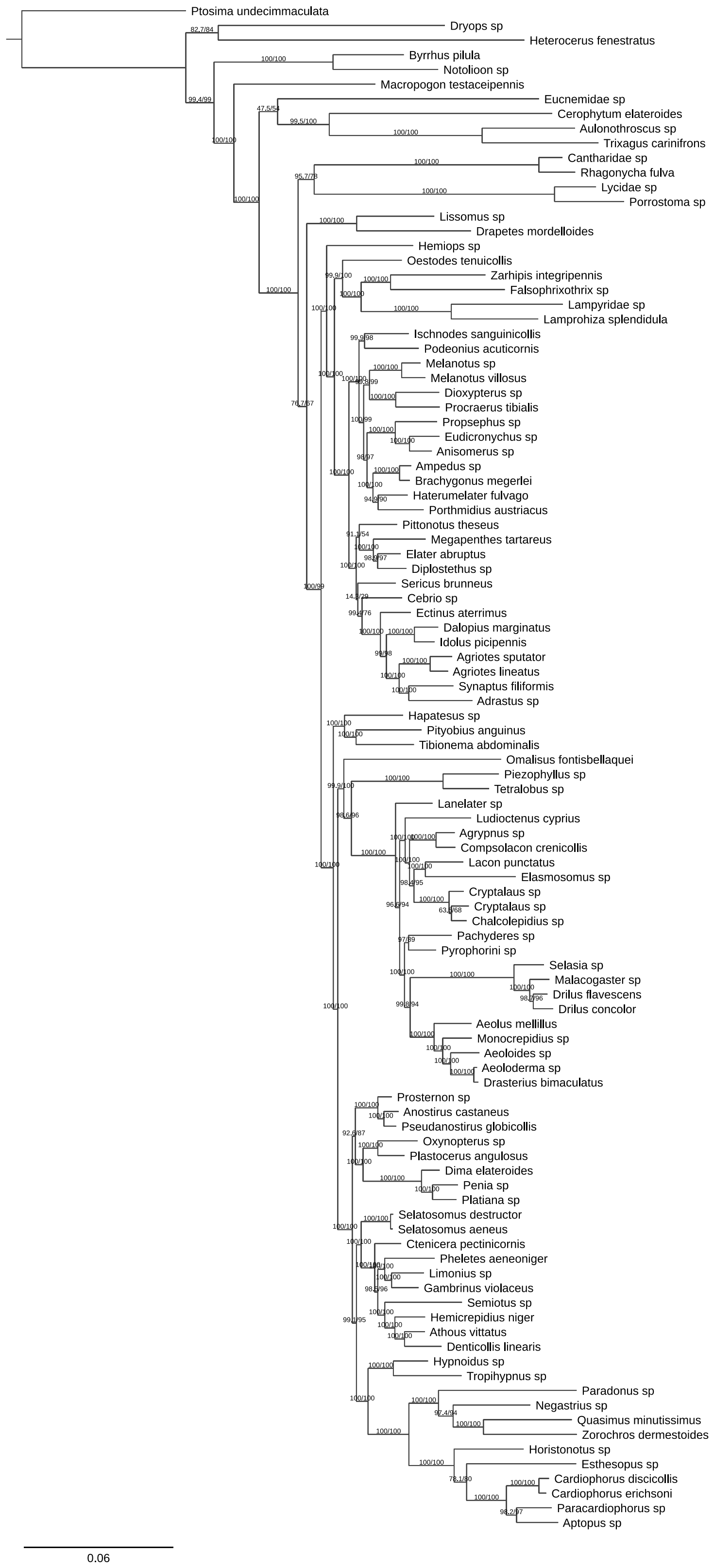

Phylogeny inferred from maximum likelihood analysis of partitioned, concatenated nucleotide data with third codon positions removed, 50% completeness matrix (50CP-No3)

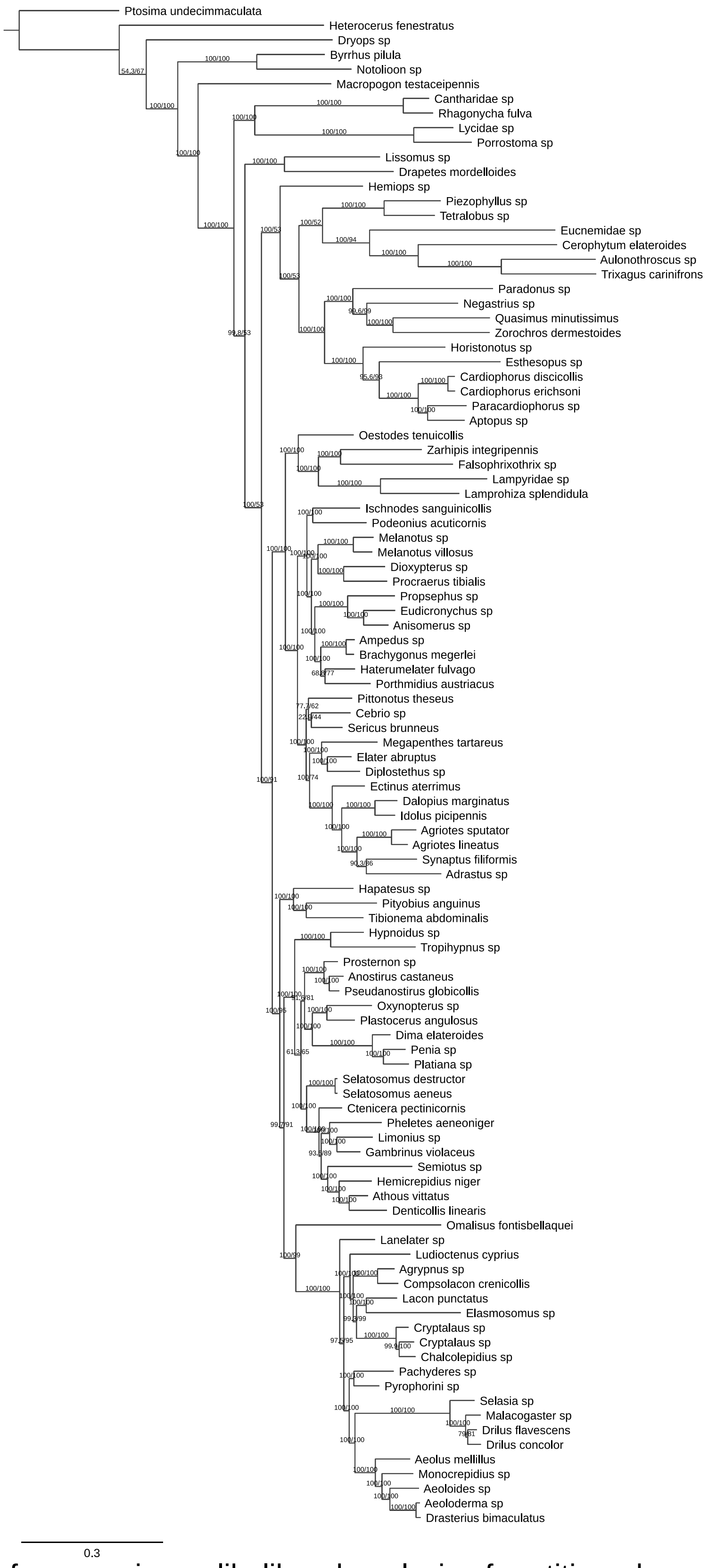

Phylogeny inferred from maximum likelihood analysis of partitioned, concatenated nucleotide data with flanking regions added, 75% completeness matrix (75CPF)

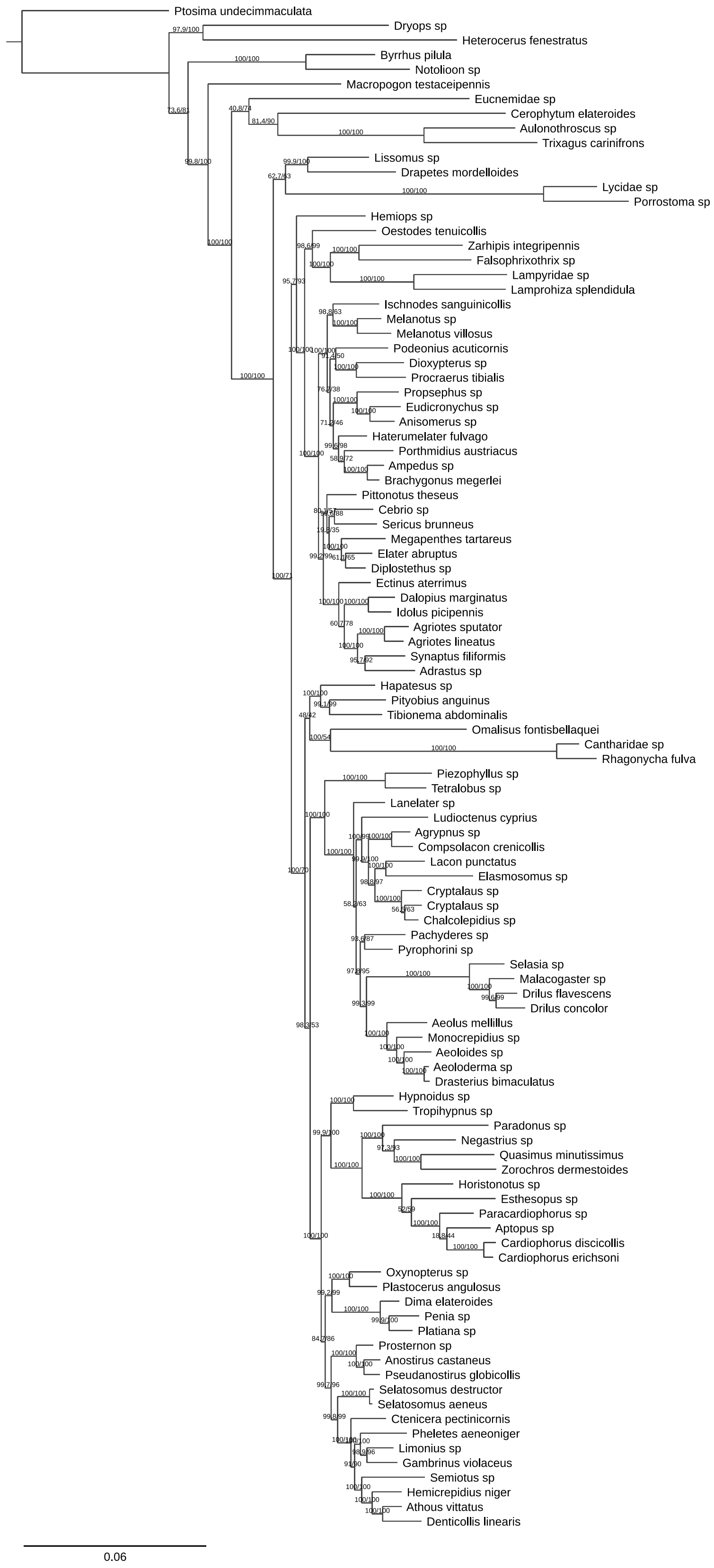

Phylogeny inferred from maximum likelihood analysis of unpartitioned, concatenated amino acid data, 75% completeness matrix (75CU-AA)

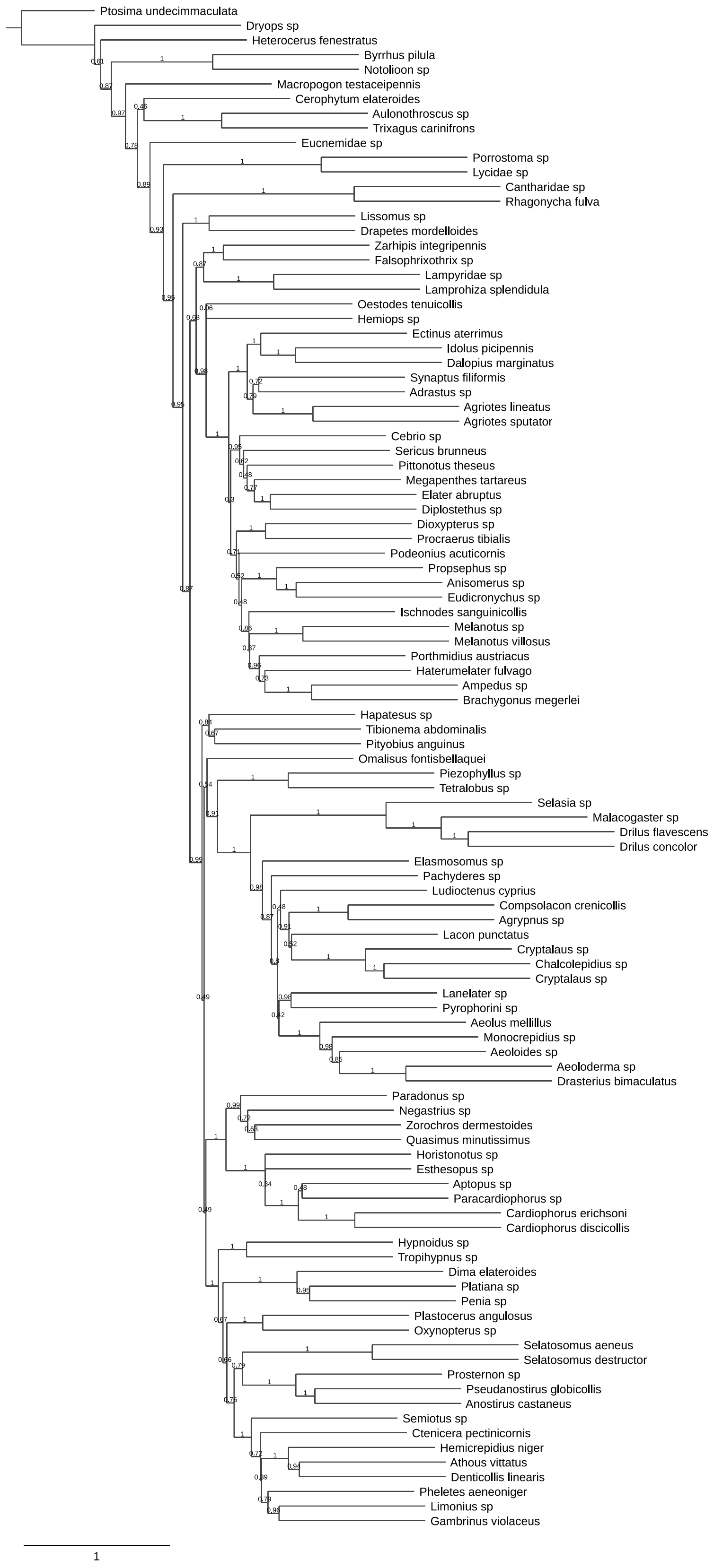

Phylogeny inferred from coalescent analysis (ASTRAL-III) of amino acid data, 50% completeness matrix (Astral50-AA)

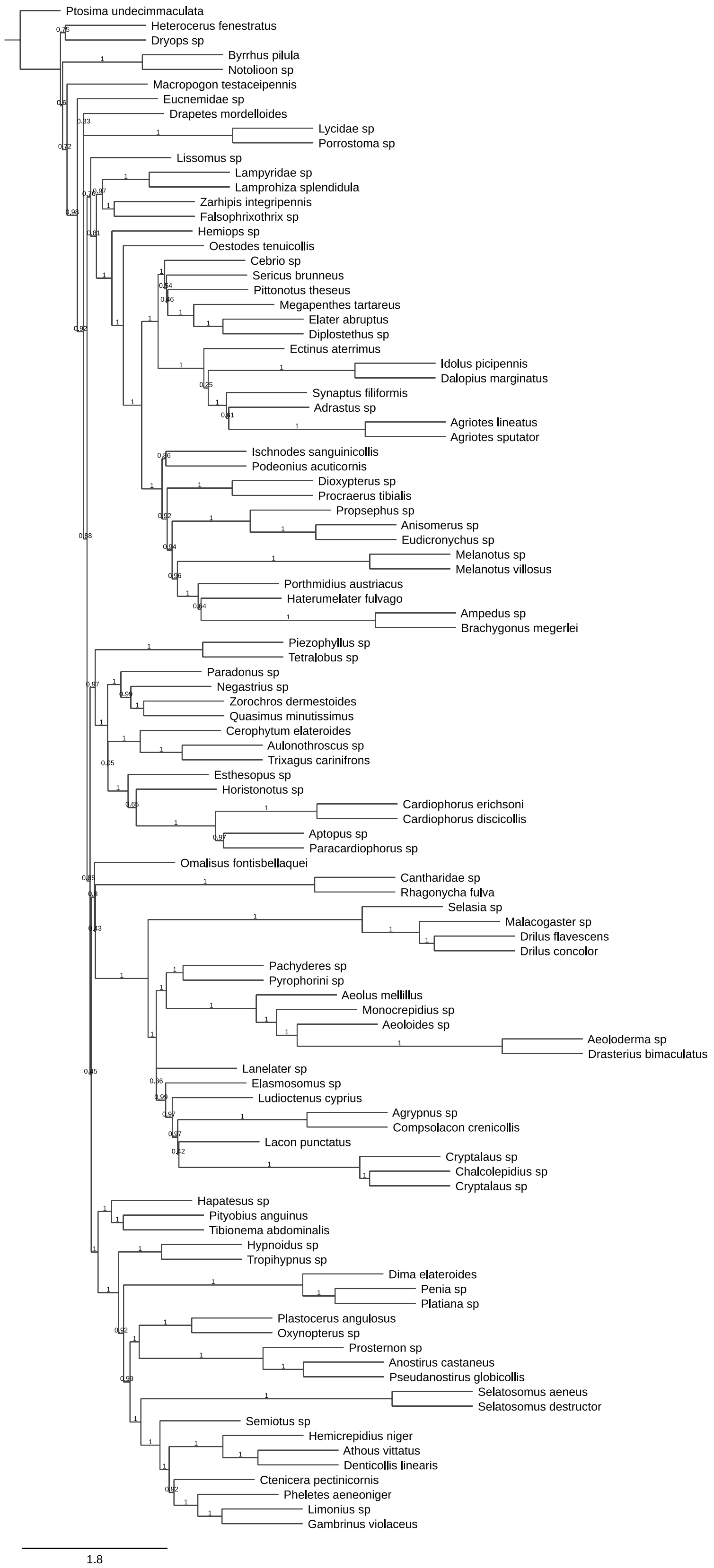

Phylogeny inferred from coalescent analysis (ASTRAL-III) of nucleotide data, 50% completeness matrix (Astral50)
